# Modelling and Predicting eHealth Usage in Europe: A Multidimensional Approach From an Online Survey of 13,000 European Union Internet Users

**DOI:** 10.2196/jmir.5605

**Published:** 2016-07-22

**Authors:** Joan Torrent-Sellens, Ángel Díaz-Chao, Ivan Soler-Ramos, Francesc Saigí-Rubió

**Affiliations:** ^1^ Department of Economics and Business Universitat Oberta de Catalunya Barcelona Spain; ^2^ Applied Economics Department Rey Juan Carlos University Madrid Spain; ^3^ Department of Health Science Universitat Oberta de Catalunya Barcelona Spain

**Keywords:** Internet, eHealth usage, health care, health drivers, health barriers, health attitude, health information, health empowerment, information and communication technologies, structural equation modelling, Europe

## Abstract

**Background:**

More advanced methods and models are needed to evaluate the participation of patients and citizens in the shared health care model that eHealth proposes.

**Objective:**

The goal of our study was to design and evaluate a predictive multidimensional model of eHealth usage.

**Methods:**

We used 2011 survey data from a sample of 13,000 European citizens aged 16–74 years who had used the Internet in the previous 3 months. We proposed and tested an eHealth usage composite indicator through 2-stage structural equation modelling with latent variables and measurement errors. Logistic regression (odds ratios, ORs) to model the predictors of eHealth usage was calculated using health status and sociodemographic independent variables.

**Results:**

The dimensions with more explanatory power of eHealth usage were health Internet attitudes, information health Internet usage, empowerment of health Internet users, and the usefulness of health Internet usage. Some 52.39% (6811/13,000) of European Internet users’ eHealth usage was more intensive (greater than the mean). Users with long-term health problems or illnesses (OR 1.20, 95% CI 1.12–1.29) or receiving long-term treatment (OR 1.11, 95% CI 1.03–1.20), having family members with long-term health problems or illnesses (OR 1.44, 95% CI 1.34–1.55), or undertaking care activities for other people (OR 1.58, 95% CI 1.40–1.77) had a high propensity toward intensive eHealth usage. Sociodemographic predictors showed that Internet users who were female (OR 1.23, 95% CI 1.14–1.31), aged 25–54 years (OR 1.12, 95% CI 1.05–1.21), living in larger households (3 members: OR 1.25, 95% CI 1.15–1.36; 5 members: OR 1.13, 95% CI 0.97–1.28; ≥6 members: OR 1.31, 95% CI 1.10–1.57), had more children <16 years of age (1 child: OR 1.29, 95% CI 1.18–1.14; 2 children: OR 1.05, 95% CI 0.94–1.17; 4 children: OR 1.35, 95% CI 0.88–2.08), and had more family members >65 years of age (1 member: OR 1.33, 95% CI 1.18–1.50; ≥4 members: OR 1.82, 95% CI 0.54–6.03) had a greater propensity toward intensive eHealth usage. Likewise, users residing in densely populated areas, such as cities and large towns (OR 1.17, 95% CI 1.09–1.25), also had a greater propensity toward intensive eHealth usage. Educational levels presented an inverted U shape in relation to intensive eHealth usage, with greater propensities among those with a secondary education (OR 1.08, 95% CI 1.01–1.16). Finally, occupational categories and net monthly income data suggest a higher propensity among the employed or self-employed (OR 1.07, 95% CI 0.99–1.15) and among the minimum wage stratum, earning ≤€1000 per month (OR 1.66, 95% CI 1.48–1.87).

**Conclusions:**

We provide new evidence of inequalities that explain intensive eHealth usage. The results highlight the need to develop more specific eHealth practices to address different realities.

## Introduction

In recent years, there has been considerable development in the field of eHealth services. With eHealth, a wide range of new opportunities has emerged to improve people’s health status through the use of information and communication technologies (ICTs) in general and the Internet in particular [1-3]. In the current context of severe constraints on health budgets, eHealth is becoming a very useful instrument to improve equality of access to, and the quality of, health care [4]. However, despite being widely used and having different characteristics depending on its application, eHealth has not been precisely defined. It is an emergent practice at the intersection of medical informatics, public health, and business [3]. In the face of this conceptual limitation, several important contributions have been made in the literature. Oh et al [5] compared 51 definitions of eHealth, and van Gemert-Pijnen et al [6] identified 16 eHealth frameworks based on their theoretical antecedents, their different visions, and the strategies or principles for increasing the uptake and impact of eHealth technologies. However, the most commonly cited definition on the Internet is Eysenbach’s [3] and it constituted the starting point of our study.

With new developments in wireless technologies, Web 2.0, and Media 3.0, eHealth has continued to profoundly change health care, which is shifting from an individual approach (care of acute health problems) toward a population approach (disease prevention and management through online communities) [7]. Consequently, health care provision models are evolving in a way that empowers patients to take care of, and make decisions on, their health [7]. Access to a wide range of health information, which used to be hard for the general public to obtain [8,9], and the sharing and posting of user content or comments in blogs and videos [10] have also been identified as means to enable greater patient empowerment and better self-care [11]. Today, patient-centered health care is recognized as the cornerstone of health care systems because it allows for improvements in health care outcomes and quality [12] to be made by reducing costs [13] and resource usage [14]. More and more patients are now better prepared for (they have the necessary knowledge to make decisions) and more informed about a wide range of health care-related topics [15-17]. They want to use ICTs in general and the Internet in particular to communicate with each other and share personal information about health [18,19].

In the context and objectives of the digital agenda for Europe, the eHealth Action Plan 2012–2020 promotes patient-centered care, thereby empowering citizens to make health decisions [20]. The aim is to foster the sustainability and efficiency of European health care systems by unlocking innovation and promoting changes in health care organizations. However, there is still very little consensus on exactly what the implications are of getting patients and citizens involved in this shared health care model [21,22], on how eHealth technologies match users’ anticipated needs [18,23,24], or, indeed, on what the main indicators of participation should be or how they should be measured [25]. It is therefore very difficult to compare the results obtained [26,27]. Obtaining empirical evidence of inequalities in health Internet usage is a work in progress [26,28,29], and not all studies consider the necessary variables [28,30,31] or are suitably adapted to factors that could foster health Internet usage in a constantly changing digital environment [26,27]. In addition, research on health Internet usage as a whole is still very scant in Europe [32] because most of the literature comes from the United States.

Since any impact fluctuates over time and in a given context [33-35], it has been suggested in the literature that there is a need to use more advanced methods to evaluate the participation of patients and citizens in this shared health care model. Social theory [36] points out that the analysis of health Internet usage disparities requires a more integrated approach that takes into account the drivers and barriers presented by the characteristics of people, of socioeconomic and cultural environments, and of technology usage [37,38]. Among other dimensions having an impact on health Internet usage, the sex and age of patients and citizens [27,39,40] have been noted, as have sociodemographic factors such as education or literacy [41,42], health status [28,39,40,43,44], and psychographic indicators such as the trust that people place in the Internet, in their own physicians, or in the health care system. Only a comprehensive examination of these dimensions will facilitate a better understanding of the complexity of citizens’ and patients’ eHealth usage [6]. Indeed, citizens’ and patients’ lack of knowledge of eHealth-related opportunities and challenges has already been identified in the eHealth Action Plan 2012–2020 as the main barrier to the acceptance of eHealth solutions in Europe [20].

Thus, the main aim of this work was to model and predict eHealth usage in Europe. We designed a multidimensional model for this purpose. The model has 9 dimensions and 88 indicators. We constructed an eHealth usage composite indicator by means of a structural equation modelling (SEM) analysis of a sample of 13,000 European Internet users in 2011. We then conducted a study to establish the indicator’s main predictors, especially the Internet users’ sociodemographic variables and health status. The results obtained provide new evidence of eHealth usage in Europe and have implications for the design of public health policies.

## Methods

### Participants and Procedure

Data for this study were drawn from the Strategic Intelligence Monitor on Personal Health Systems Phase 2 (SIMPHS2) research project “Citizens and ICT for health in 14 European countries: results from an online panel” [45]. The study was carried out by the Institute for Prospective Technological Studies in cooperation with the European Commission Directorate General for Information Society and Media, now the Directorate General for Communications Networks, Content and Technology. The SIMPHS2 citizen panel survey’s analysis of user demand had as its main objectives (1) to develop typologies of digital health care users and measure the impact of ICT and the Internet on health status, health care demand, and health management, and (2) to identify factors that can enhance or inhibit the role and use of personal health systems from a citizen’s perspective with special emphasis on mHealth, remote patient monitoring and treatment disease management, telecare, telemedicine, and wellness [45].

Our study used survey data for a sample of 13,000 European citizens aged 16–74 years who had used the Internet in the previous 3 months (Multimedia Appendix 1). The sampling universe comprised 171,859,356 European citizens aged 16–74 years with an overall margin of error of ±0.88 in the case of maximum indetermination p=q=50%, for a confidence level of 95.5%. The sample had two essential characteristics. First, we chose an equal-sized sample for each of the 13 countries being studied, that is to say, 1000 interviews for each country in the sample: Austria, Belgium, Denmark, Estonia, Finland, France, Germany, Italy, the Netherlands, Sweden, Slovakia, Slovenia, and Spain (public data are available for 13 countries). The country-specific margin of error was ±3.16 in the case of maximum indetermination p=q=50%, for a confidence level of 95.5%. Second, we chose to use a fully representative sample for the distribution of the target population, according to sex and age group. The demographic groups are organized by the cross-referenced quotas of sex and age group, as follows: women aged 16–24 years (±2.78), women aged 25–54 years (±1.58), women aged 55–74 years (±3.08), men aged 16–24 years (±2.73), men aged 25–54 years (±1.56), and men aged 55–74 years (±2.89).

The questionnaire used in the survey contained 47 questions grouped into 5 dimensions (Multimedia Appendix 2): (1) health status, and health care and social care services use (12 questions), (2) health attitude and health information sources (5 questions) (3) Internet and ICT uses (2 questions), (4) health-related use of ICTs and the Internet (15 questions), and (5) sociodemographic profile of participants (13 questions). The survey was answered by European Internet users in online interviews, lasting for half an hour each, and in a native language of each country. A study presentation paragraph was written to inform potential respondents about the confidentiality of any data provided and the academic aim of the research. The European Internet users voluntarily answered the questionnaire and did not receive any payment in cash or kind. While the questionnaire was being implemented, an expert was on hand at all times (by email) to resolve any queries that the respondents had. The respondent citizens were selected by means of probability sampling applied to each country universe. The net response rate was 20.72%. To achieve 13,000 responses, it was necessary to send 65,126 invitations, to which 19,731 responses were received. Of the responses received, 6731 were excluded, either because they did not fall into the required quotas for Internet use (6236) or because they had been rejected (495). The reasons for rejecting a questionnaire were either that they were incomplete or that the consistency of responses was poor. The fieldwork period ran from the July 20 to August 20, 2011.

The SIMPHS2 research project followed the Checklist for Reporting Results of Internet E-Surveys criteria [46]. For a more detailed explanation, see the SIMPHS2 research report [45].

### Data Analysis and Models

From an empirical perspective, explanatory factors determining eHealth usage raise two particular difficulties. First, the approach to the concept requires a multidimensional basis that is not usually captured in a single variable. In fact, the most common approaches found in the literature perform partial analyses of its various dimensions. This type of analysis has the disadvantage of not taking a full snapshot of the explanatory factors, which gives rise to the second difficulty: statistical modelling. In other words, eHealth usage can be interpreted as a latent, nonobservable concept, which therefore calls for statistical techniques that allow variables of this type, which are not directly measurable, to be used [47,48].

In the empirical literature, SEM with latent variables has been used to overcome this problem. A general SEM is a formal mathematical model. It is a set of linear equations that encompasses various types of models, such as regression analysis models, simultaneous equation systems, factor analysis, and path analysis. The main advantage of this method of analysis is the incorporation of different types of variables into the SEM. Directly observable and measurable variables, and theoretical or latent variables representing concepts that are not directly observed can therefore be incorporated. When the variable to be explained (dependent) is latent, it must be continuous, whereas dependent observed variables can be continuous, censored, binary, ordered, or categorical (ordinals), or combinations of any of these variable types [49].

This method of analysis allows us to define eHealth usage as a latent variable, thus enabling us to calculate the specific explanatory effect of the variables that it comprises. Hence, besides building an overall explanatory model of the determinants of eHealth usage, it is also possible to identify which of its explanatory dimensions are more important. In addition, SEM enables the relationships between the different observable variables included in the model (indirect effects) to be estimated. In this initial approach, however, only the direct effects are presented, that is to say, the coefficients of causality between the individual indicators and their latent dimensions, and later between the estimated dimensions and the latent variable (eHealth usage). In this context, and in order to capture the factors that explain eHealth usage in a large sample of European Internet users, we proposed and tested a 2-stage SEM with latent variables and measurement errors for 2011.

We applied the 2-stage empirical estimation methodology as follows: in the first stage, we tested the causal relationships among 88 indicators and the 9 latent dimensions describing eHealth usage in Europe, and in the second stage, we tested the causal relationships among the indicators constructed for those 9 dimensions (based on the coefficients from the first stage) and the latent construct of eHealth usage. Finally, after applying the coefficients obtained from the second stage, we constructed an eHealth usage indicator and determined its mean values (total and for the 9 dimensions). This methodology involved the design and statistical testing of 10 empirical models: 9 models for the first stage and 1 model for the second stage.

**Figure 1:**
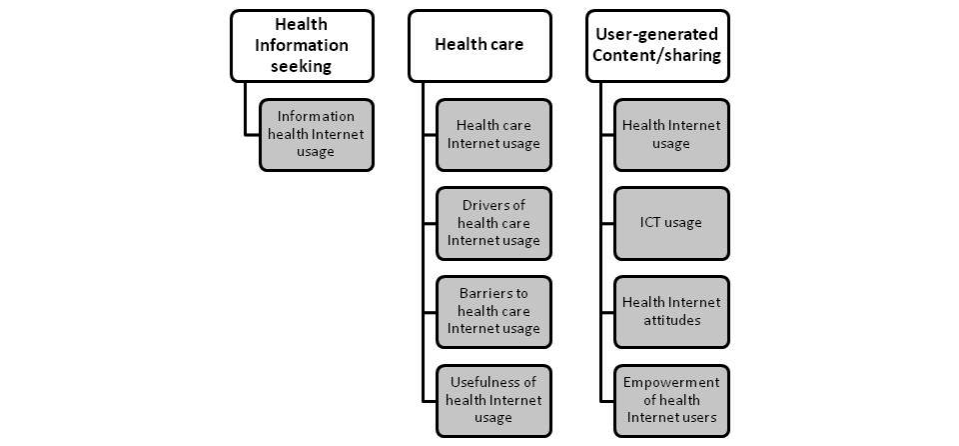
Flow diagram detailing the multidimensional model of eHealth usage. ICT: information and communication technologies.

Several eHealth definitions highlight growing patient empowerment (access to information and ability to use it) and point to the potential of eHealth to facilitate doctor-patient communication, partnership, and shared decision making [3,41,50]. Figure 1 shows the multidimensional model of eHealth usage with 9 dimensions grouped in 3 domains relevant to health usage: health information seeking, health care, and user-generated content and sharing. The 9 explanatory model dimensions and variables are as follows: *dimension 1: health Internet usage*, captured by a set of 14 variables measuring the frequency of usage (Multimedia Appendix 3); *dimension 2: health care Internet usage*, captured by a set of 10 variables measuring the frequency of usage (Multimedia Appendix 4); *dimension 3: drivers of health care Internet usage*, captured by a set of 8 variables measuring the factors that Internet users consider relevant when evaluating an Internet health site (Multimedia Appendix 5); *dimension 4: barriers to health care Internet usage*, captured by a set of 10 variables measuring the factors that Internet users regarded as barriers when evaluating Internet health care (Multimedia Appendix 6); *dimension 5: usefulness of health Internet usage*, captured by a set of 13 variables measuring the Internet user’s perceived usefulness of health Internet usage (Multimedia Appendix 7); *dimension 6: ICT usage*, captured by a set of 15 variables measuring the frequency of usage (Multimedia Appendix 8); *dimension 7: information health Internet usage*, captured by a set of 7 variables measuring the Internet user’s perceived judgment of information health usage (Multimedia Appendix 9); *dimension 8: health Internet attitudes*, captured by a set of 6 variables measuring the Internet user’s perceived feelings about health Internet usage (Multimedia Appendix 10); *dimension 9: empowerment of health Internet users*, captured by a set of 5 variables measuring the Internet user’s perceived results of health Internet usage (Multimedia Appendix 11).

Additionally, we calculated the logistic regression to model the predictors of eHealth usage using health status and sociodemographic independent variables. For each independent variable, we calculated odds ratios (ORs) and their 95% CIs. We used IBM SPSS Amos v.22 (IBM Corp) for all calculations.

## Results

### eHealth Usage Composite Indicator

Table 1 shows the results (standardized coefficients and measurement errors) of the first stage of estimating the explanatory factors of eHealth usage in Europe in 2011. In this first stage, we estimated the causal relationships among 88 indicators and the 9 dimensions describing eHealth usage by using an SEM with measurement errors. First, it should be noted that all the variables specified in the model were statistically significant (99% confidence level). Second, the goodness-of-fit measurements for the 9 proposed models were highly satisfactory. Thus, the normed fit index (NFI), relative fit index (RFI), incremental fit index (IFI), Tucker-Lewis index (TLI), and comparative fit index (CFI) had very high values, approaching the optimal value of 1. The root mean square error of approximation (RMSEA) values were <0.08, thus corroborating the validity of the estimated models.

**Table 1:** Explanatory factors of eHealth usage in Europe (first stage)^a^ in 2011.

Dimension/variable			Standardized coefficient	*P* value	Error	*P* value
**1. Health Internet usage**					0.698	<.001
	1.	Look for information about a physical illness	0.536	<.001	1.732	<.001
	2.	Look for information about wellness or lifestyle	0.545	<.001	1.955	<.001
	3.	Buy medicine or vitamins online	0.779	<.001	2.558	<.001
	4.	Participate in an online support group with people	0.774	<.001	2.761	<.001
	5.	Participate in social networking sites	0.790	<.001	2.301	<.001
	6.	Use email or Web to communicate with a doctor’s office	0.713	<.001	3.301	<.001
	7.	Click on a health or medical Web’s privacy policy	0.682	<.001	3.750	<.001
	8.	Describe a medical condition to get advice from an online doctor	0.783	<.001	2.645	<.001
	9.	Describe a medical condition to get advice from other online users	0.822	<.001	1.905	<.001
	10.	Bookmark or favorite a health website	0.725	<.001	2.426	<.001
	11.	Look to see what company is providing the information on a health website	0.681	<.001	2.661	<.001
	12.	Look for information about a mental health issue	0.749	<.001	2.209	<.001
	13.	Disclose medical information on social networking sites	0.821	<.001	2.329	<.001
	14.	Disclose medical information on websites to share files	0.814	<.001	2.516	<.001
	Goodness-of-fit indexes: NFI^b^: 0.986; RFI^c^: 0.979; IFI^d^: 0.987; TLI^e^: 0.980; CFI^f^: 0.987; RMSEA^g^: 0.041					
**2. Health care Internet usage**					1.982	<.001
	15.	Make an Internet appointment with health care professionals	0.743	<.001	1.609	<.001
	16.	Receive an email from doctor, nurse, or health care organization	0.781	<.001	1.343	<.001
	17.	Have an online consultation through videoconference with health care professionals	0.813	<.001	1.675	<.001
	18.	Receive online the results of clinical or medical test	0.801	<.001	1.484	<.001
	19.	Use medical information through an Internet provider	0.776	<.001	2.098	<.001
	20.	Use medical information through an Internet health care organization	0.812	<.001	1.656	<.001
	21.	Use a game console to play games related to health or wellness	0.739	<.001	2.056	<.001
	22.	Use a health/wellness app on mobile phone	0.790	<.001	1.643	<.001
	23.	Use electronic devices to transmit clinical or medical information	0.758	<.001	1.811	<.001
	24.	Email about health promotion or health prevention	0.670	<.001	1.906	<.001
	Goodness-of-fit indexes: NFI: 0.971; RFI: 0.953; IFI: 0.971; TLI: 0.954; CFI: 0.971; RMSEA: 0.074									
**3. Drivers of health care Internet usage**					0.237	<.001
	25.	Secure handling of personal information	0.672	<.001	0.287	<.001
	26.	Information in own language	0.580	<.001	0.407	<.001
	27.	Updated information	0.737	<.001	0.246	<.001
	28.	Interactivity	0.520	<.001	0.579	<.001
	29.	Involvement of health professionals	0.867	<.001	0.150	<.001
	30.	Clear statement of who is responsible for sponsoring the site	0.586	<.001	0.614	<.001
	31.	Involvement of health organizations	0.728	<.001	0.322	<.001
	32.	Involvement of governments	0.382	<.001	0.794	<.001
	Goodness-of-fit indexes: NFI: 0.973; RFI: 0.934; IFI: 0.973; TLI: 0.935; CFI: 0.973; RMSEA: 0.075					
**4. Barriers to health care Internet usage**					0.296	<.001
	33.	Lack of digital skills	0.583	<.001	0.574	<.001
	34.	Lack of access to ICT^h^ for health applications	0.632	<.001	0.452	<.001
	35.	Lack of motivation and interest	0.666	<.001	0.382	<.001
	36.	Lack of awareness	0.730	<.001	0.329	<.001
	37.	Lack of health literacy	0.714	<.001	0.352	<.001
	38.	Lack of trust	0.832	<.001	0.199	<.001
	39.	Lack of liability	0.810	<.001	0.242	<.001
	40.	Lack of privacy	0.762	<.001	0.279	<.001
	41.	Lack of security	0.800	<.001	0.232	<.001
	42.	Lack of reliability	0.804	<.001	0.219	<.001
	Goodness-of-fit indexes: NFI: 0.979; RFI: 0.953; IFI: 0.980; TLI: 0.953; CFI: 0.980; RMSEA: 0.074					
**5. Usefulness of health Internet usage**					0.720	<.001
	43.	ICT for health could increase other ICT uses	0.751	<.001	0.555	<.001
	44.	ICT for health could lead to greater patient satisfaction	0.819	<.001	0.372	<.001
	45.	ICT for health could improve health status	0.782	<.001	0.471	<.001
	46.	ICT for health could improve the ability to take care of one’s own health	0.816	<.001	0.385	<.001
	47.	ICT for health could change behaviors toward a healthy lifestyle	0.769	<.001	0.469	<.001
	48.	ICT for health could avoid travelling expenses and time	0.740	<.001	0.567	<.001
	49.	ICT for health could improve the quality of health care services	0.803	<.001	0.407	<.001
	50.	Internet health could substitute for offline consultations with the physicians	0.604	<.001	1.022	<.001
	51.	Internet health complements offline consultations with the physicians	0.704	<.001	0.687	<.001
	52.	Quality of Internet health is aligned with the quality of offline services	0.626	<.001	0.796	<.001
	53.	Personal information could be shared with physicians through Internet due to privacy	0.273	<.001	1.202	<.001
	54.	Patients could be more comfortable with a remote monitoring system to track health	0.626	<.001	0,895	<.001
	55.	Patients could be willing to pay to access Internet health services	0.512	<.001	1.140	<.001
	Goodness-of-fit indexes: NFI: 0.979; RFI: 0.953; IFI: 0.980; TLI: 0.953; CFI: 0.980; RMSEA: 0.074					
**6. ICT usage**					0.037	<.001
	56.	Use a search engine to find information	0.242	<.001	0.600	<.001
	57.	Send emails with attached files	0.344	<.001	0.987	<.001
	58.	Post messages to chatrooms, newsgroups, or an online discussion forum	0.626	<.001	1.290	<.001
	59.	Use the Internet to make telephone calls	0.520	<.001	1.377	<.001
	60.	Use peer-to-peer file sharing for exchanging pictures, videos, or movies	0.637	<.001	1.042	<.001
	61.	Create a webpage	0.552	<.001	0.856	<.001
	62.	Use websites to share pictures, videos, or movies	0.681	<.001	1.103	<.001
	63.	Use a social networking site	0.436	<.001	1.972	<.001
	64.	Purchase goods or services online	0.472	<.001	0.801	<.001
	65.	Keep a blog or weblog	0.564	<.001	0.939	<.001
	66.	Use instant messaging or chat websites	0.564	<.001	1.638	<.001
	67.	Do home banking	0.184	<.001	1.577	<.001
	68.	Use online software	0.612	<.001	1.230	<.001
	69.	Use the Internet through mobile phone	0.523	<.001	1.833	<.001
	70.	Use online gaming or playing games console	0.371	<.001	1.976	<.001
	Goodness-of-fit indexes: NFI: 0.942; RFI: 0.912; IFI: 0.944; TLI: 0.914; CFI: 0.944; RMSEA: 0.051					
**7. Information health Internet usage**					0.656	<.001
	71.	Better informed about the advice of the health care professionals	0.792	<.001	0.389	<.001
	72.	Better understanding of personal health	0.830	<.001	0.301	<.001
	73.	Better informed on what is available, so that can make own choices	0.802	<.001	0.341	<.001
	74.	Better understand the relevance of personal health	0.817	<.001	0.323	<.001
	75.	Know more about the opinions of people who are in similar situations	0.708	<.001	0.505	<.001
	76.	Better understand personal health through online discussions or experiences	0.733	<.001	0.524	<.001
	77.	Play a more active role in exchanges with health care professionals	0.728	<.001	0.547	<.001
	Goodness-of-fit indexes: NFI: 0.993; RFI: 0.985; IFI: 0.993; TLI: 0.986; CFI: 0.993; RMSEA: 0.046					
**8. Health Internet attitudes**					0.673	<.001
	78.	Better equipped to implement the advice of health care professionals	0.819	<.001	0.332	<.001
	79.	Better equipped to make own choices without the advice of a physician	0.791	<.001	0.433	<.001
	80.	Better equipped to make positive changes through other people	0.805	<.001	0.353	<.001
	81.	More confident in playing a more active role in relationship with physician	0.834	<.001	0.319	<.001
	82.	More confident about choices on possible treatments and solutions	0.863	<.001	0.265	<.001
	83.	More confident in discussions with the people in one’s life	0.795	<.001	0.387	<.001
	Goodness-of-fit indexes: NFI: 0.998; RFI: 0.991; IFI: 0.998; TLI: 0.992; CFI: 0.998; RMSEA: 0.041					
**9. Empowerment health Internet users**					0.665	<.001
	84.	Make decisions on health, albeit without going against the physicians	0.760	<.001	0.486	<.001
	85.	Take a more active role in health by deciding solutions or alternative approaches	0.840	<.001	0.317	<.001
	86.	Make decisions about health on the basis of own preferences	0.825	<.001	0.384	<.001
	87.	Take a more active role in health by continuing to talk with people	0.775	<.001	0.414	<.001
	88.	Make decisions about health by relying on the experiences of other people	0.783	<.001	0.452	<.001
	Goodness-of-fit indexes: NFI: 0.997; RFI: 0.988; IFI: 0.997; TLI: 0.992; CFI: 0.988; RMSEA: 0.048					

^a^Regression analysis: structural equation modelling; direct effects.

^b^NFI: normed fit index.

^c^RFI: relative fit index.

^d^IFI: incremental fit index.

^e^TLI: Tucker-Lewis index

^f^CFI: comparative fit index.

^g^RMSEA: root mean square error of approximation.

^h^ICT: information and communication technology.

In the health Internet usage dimension, the standardized coefficient variability is 0.3 points. The variables with the greatest explanatory power in this dimension are related to describing a medical condition to get advice from other Internet users (0.822), as well as disclosing medical information on social networking sites (0.821) or on websites (0.814). In contrast, less explanatory variables are related to finding information about physical illness (0.536) or wellness and lifestyle (0.545). In the health care Internet usage dimension, the standardized coefficient variability is 0.14 points, between the explanatory variables related to online consultation through videoconference with health care professionals (0.813), using medical information through an Internet health care organization (0.812), and receiving emails about health promotion or health prevention (0.670). In the drivers of health care Internet usage dimension, the standardized coefficient variability is high and reaches about 0.5 points. The variable with the greatest explanatory power is the involvement of health professionals (0.867), and the variable with the least explanatory power is the involvement of governments (0.382). In the barriers to health care Internet usage dimension, variability is 0.25 points, between the lack of trust (0.832), liability (0.810), reliability (0.804), and security (0.800) and the lack of digital skills (0.583). In the usefulness of health Internet usage dimension, the explanatory variable variability is around 0.3 points, from the perceptions that ICT for health could lead to greater patient satisfaction (0.819), could improve the ability to take care of one’s own health (0.816), and could improve the quality of health care services (0.803) to the willingness to pay to access Internet health services (0.512). In the ICT usage dimension, variability is the highest, and is around 0.5 points, from using the Internet to share pictures, videos, or movies (0.681), peer-to-peer file sharing (0.637), posting messages to chat rooms, newsgroups, or online discussion forums (0.626), and using online software (0.612) to using a search engine to find information (0.242) and home banking (0.184). Finally, in the information health Internet usage, health Internet attitudes, and empowerment of health Internet users dimensions, the explanatory variable variability is minimal, and all the obtained coefficients are in the range from 0.7 to 0.8 points.

Table 2 shows the results (standardized coefficients and measurement errors) of the second stage of estimating the explanatory factors of eHealth usage in Europe in 2011. In this second stage, we tested the causal relationships among the indicators constructed for the 9 dimensions describing eHealth usage (based on the coefficients from the first stage) and the latent construct of explanatory factors of eHealth usage by using an SEM with a latent dependent variable and measurement errors. First, it should be noted that all the variables specified in the model were statistically significant (95% confidence level, at least). Second, the goodness-of-fit measurements for the proposed model were highly satisfactory. Thus, the indexes NFI (0.981), RFI (0.961), IFI (0.981), TLI (0.962), and CFI (0.981) had very high values, approaching the optimal value of 1. The RMSEA value was <0.08 (0.052), thus corroborating the validity of the estimated model.

**Table 2:** Explanatory factors of eHealth usage in Europe (second stage)^a^ in 2011.

Dimension/variable			Standardized coefficient	*P* value	Error	*P* value
**eHealth usage**					3.538	<.001
	1.	Health Internet usage	0.099	<.001	360.143	<.001
	2.	Health care Internet usage	0.029	<.001	161.145	<.001
	3.	Drivers of health care Internet usage	0.311	<.001	8.003	<.001
	4.	Barriers to health care Internet usage	0.221	<.001	21.665	<.001
	5.	Usefulness of health Internet usage	0.547	<.001	37.930	<.001
	6.	Information and communication technology usage	0.240	<.001	31.880	<.001
	7.	Information health Internet usage	0.859	<.001	5.221	<.001
	8.	Health Internet attitudes	0.940	<.001	2.146	<.001
	9.	Empowerment of health Internet users	0.855	<.001	3.446	<.001
	Goodness-of-fit indexes: NFI^b^: 0.981; RFI^c^: 0.961; IFI^d^: 0.981; TLI^c^: 0.962; CFI^f^: 0.981; RMSEA^g^: 0.053									

^a^Regression analysis: structural equation modelling; estimated coefficients: direct effects.

^b^NFI: normed fit index.

^c^RFI: relative fit index.

^d^IFI: incremental fit index.

^e^TLI: Tucker-Lewis index

^f^CFI: comparative fit index.

^g^RMSEA: root mean square error of approximation.

The standardized coefficients obtained for the indicators of the 9 dimensions of eHealth usage in Europe highlight different explanatory capabilities. The dimensions with more-explanatory power are health Internet attitudes (0.940), information health Internet usage (0.859), empowerment of health Internet users (0.855), and usefulness of health Internet usage (0.547). ICT usage (0.240), and drivers of (0.311) and barriers to (0.221) health care Internet usage fall in the middle. Finally, the health Internet usage (0.099) and health care Internet usage (0.029) standardized coefficients have the least eHealth usage explanatory power. After applying the coefficients obtained from the second stage, we constructed an eHealth usage composite indicator and determined its mean values (Table 3).

**Table 3:** eHealth usage composite indicator descriptive statistics, 2011.

Dimension/variable		Mean	SD	Minimum	Maximum	Skewness	Kurtosis
1.	Health Internet usage	25.37	19.07	10.21	91.93	1.832	2.705
2.	Health care Internet usage	14.50	12.70	7.68	69.15	2.768	7.756
3.	Drivers of health care Internet usage	16.41	2.98	5.07	20.29	–1.352	2.530
4.	Barriers to health care Internet usage	23.21	4.77	7.33	29.33	–0.963	1.141
5.	Usefulness of health Internet usage	28.99	7.37	8.83	44.13	–0.458	0.375
6.	Information and communication technology usage	19.12	5.82	7.33	36.64	0.566	–0.005
7.	Information health Internet usage	20.78	4.47	5.41	27.05	–0.870	1.180
8.	Health Internet attitudes	18.22	4.29	4.91	24.54	–0.714	0.756
9.	Empowerment of health Internet users	14.35	3.58	3.98	19.92	–0.637	0.491
eHealth usage composite indicator		80.85	14.24	24.19	117.06	–0.541	0.716

**Figure 2:**
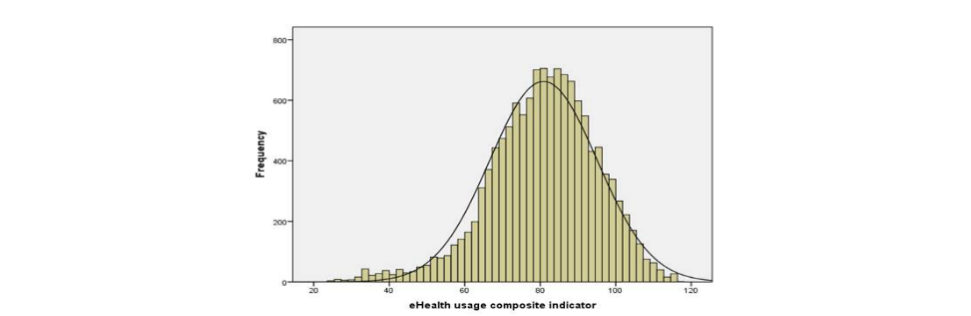
eHealth usage composite indicator histogram.

Figure 2 shows the histogram (frequencies and expected mean) of the values of the eHealth usage composite indicator. The mean value of this composite indicator was 80.85 points (SD 14.24, minimum to maximum range 24.19–117.06).

To capture the main predictors of eHealth usage in Europe, we performed a logistic regression using independent variables for European Internet users’ health status and sociodemographic circumstances. The first step in this analysis was to recode the eHealth usage composite indicator. We therefore constructed a dichotomous eHealth usage indicator, based on the mean of the composite indicator obtained. The dichotomous eHealth usage indicator takes the value 1 when the eHealth usage composite indicator is equal to or greater than the mean, and the value 0 when less than the mean. The mean value of this dichotomous composite indicator was 0.524 points (SD 0.499, minimum to maximum range 0–1, skew –0.097, kurtosis –1.991). Some 52.39% (6811/13,000) of European Internet users’ eHealth usage was more intensive (greater than the mean).

### Health Status-Related Predictors of eHealth

Table 4 shows the results of the logistic regressions (ORs) between the dichotomous eHealth usage composite indicator and the health status independent variables. We found no significant differences between European Internet users’ perceived health status and more intensive eHealth usage—the variables with the highest predictive power were poor health (OR 1.30, 95% CI 1.12–1.51) and very good health (OR 1.02, 95% CI 0.94–1.11). However, the analysis of the existence of long-term health problems or illnesses did point to its predictive power. European Internet users with long-term health problems or illnesses (OR 1.20, 95% CI 1.12–1.29) or receiving long-term treatment (OR 1.11, 95% CI 1.03–1.20) had a much greater propensity toward more intensive eHealth usage. Likewise, the existence of specific health problems or illnesses determined a greater probability of more intensive eHealth usage. Specifically, these were diabetes (OR 1.01, 95% CI 0.88–1.16), stroke or cerebral hemorrhage (OR 0.95, 95% CI 0.72–1.23), cancer (OR 0.93, 95% CI 0.77–1.12), and cataract (OR 0.91, 95% CI 0.73–1.13). In contrast, users with health problems or illnesses related to chronic bronchitis and emphysema (OR 0.69, 95% CI 0.59–0.79) and osteoporosis (OR 0.63, 95% CI 0.51–0.77) had a lower propensity toward intensive eHealth usage. Finally, having family members with or caring for other people with long-term illnesses determined a greater propensity toward more intensive eHealth usage. Users with family members having long-term health problems or illnesses (OR 1.44, 95% CI 1.34–1.55) or who cared for other people with long-term health problems or illnesses (OR 1.58, 95% CI 1.40–1.77) had a greater propensity toward more intensive eHealth usage than users without such problems.

**Table 4:** Logistic regression models for odds of dichotomous eHealth usage composite indicator reporting a value of 1 (eHealth usage composite indicator greater than or equal to eHealth usage composite indicator mean) by health status, 2011.

		OR^a^	95% CI
**Perceived general health**			
	Very poor health	0.91	0.61–1.34
	Poor health	1.30	1.12–1.51
	Neither good nor poor health	0.99	0.91–1.10
	Good health	0.94	0.88–1.01
	Very good health	1.02	0.94–1.11
**Long-standing illness or health problem**					
	Yes	1.20	1.12–1.29
	No	0.83	0.77–0.89
**Long-term medical treatment**			
	Yes	1.11	1.03–1.20
	No	0.90	0.84–0.97
**Specific illness or health problem**			
	Diabetes	1.01	0.88–1.16
	Allergy	0.82	0.77–0.88
	Asthma	0.87	0.78–0.98
	Hypertension	0.86	0.79–0.94
	Long-standing muscular problem	0.78	0.72–0.85
	Cancer	0.93	0.77–1.12
	Cataract	0.91	0.73–1.13
	Migraine or frequent headache	0.83	0.77–0.90
	Chronic bronchitis, emphysema	0.69	0.59–0.79
	Osteoporosis	0.63	0.51–0.77
	Stroke, cerebral hemorrhage	0.95	0.72–1.23
	Peptic, gastric, or duodenal ulcer	0.78	0.68–0.91
	Chronic anxiety or depression	0.72	0.66–0.79
**Family members with long-term illness or disability**					
	Yes	1.44	1.34–1.55
	No	0.69	0.65–0.75
**Taking care of a person with long-term illness or disability**					
	Yes	1.58	1.40–1.77
	No	0.64	0.57–0.71

^a^OR: odds ratio.

### Sociodemographic-Related Predictors of eHealth

Table 5 shows the results of the logistic regressions (ORs) between the dichotomous eHealth usage composite indicator and the sociodemographic independent variables. European Internet users who were female (OR 1.23, 95% CI 1.14–1.31) and who were aged 25–54 years (OR 1.12, 95% CI 1.05–1.21) had a greater propensity toward intensive eHealth usage than men (OR 0.82, 95% CI 0.76–0.88) or those in other age groups: 16–24 years (OR 0.97, 95% CI 0.89–1.06) and 55–74 years (OR 0.86, 95% CI 0.78–0.94). Households with more members (3 members: OR 1.25, 95% CI 1.15–1.36; 5 members: OR 1.13, 95% CI 0.97–1.28; ≥6 members: OR 1.31, 95% CI 1.10–1.57), more children <16 years of age (1 child: OR 1.29, 95% CI 1.18–1.41; 2 children: OR 1.05, 95% CI 0.94–1.17; 4 children: OR 1.35, 95% CI 0.88–2.08), and more members >65 years of age (1 member: OR 1.33, 95% CI 1.18–1.50; ≥4 members: OR 1.82, 95% CI 0.54–6.03) also had greater probabilities of more intensive eHealth usage.

**Table 5:** Logistic regressions models for odds of dichotomous eHealth usage composite indicator reporting a value of 1 (eHealth usage composite indicator greater than or equal to eHealth usage composite indicator mean) by sociodemographic conditions, 2011.

		OR^a^	95% CI
**Sex**			
	Male	0.82	0.76–0.88
	Female	1.23	1.14–1.31
**Age range (years)**			
	16–24	0.97	0.89–1.06
	25–54	1.12	1.05–1.21
	55–74	0.86	0.78–0.94
**Number of members in the household**			
	1	0.75	0.69–0.83
	2	0.87	0.81–0.94
	3	1.25	1.15–1.36
	4	1.07	0.98–1.16
	5	1.13	0.97–1.28
	≥6 or more	1.31	1.10–1.57
**Number of children <16 years old in the household**			
	0	0.82	0.77–0.88
	1	1.29	1.18–1.41
	2	1.05	0.94–1.17
	3	0.97	0.79–1.20
	4	1.35	0.88–2.08
	≥5	0.77	0.34–1.71
**Number of members >65 years old in the household**			
	0	0.84	0.76–0.92
	1	1.33	1.18–1.50
	2	0.97	0.84–1.14
	3	0.99	0.44–2.24
	≥4	1.82	0.54–6.03
**Country of citizenship**			
	National of 13 sample countries	0.78	0.68–0.91
	National of other EU^b^ member state	1.28	1.09–1.50
	National of non-EU country	1.25	0.90–1.73
**Country of birth**			
	Native of 13 sample countries	1.02	0.89–1.17
	Native of other EU member state	0.80	0.67–0.95
	Native of non-EU country	1.31	1.06–1.61
**Type of locality**			
	Densely populated area (cities and large towns)	1.17	1.09–1.25
	Intermediate area (towns)	0.92	0.86–0.99
	Thinly populated area (villages and rural)	0.90	0.83–0.97
**Completed level of education**			
	Primary or lower secondary education	0.87	0.80–0.95
	Upper secondary education	1.08	1.01–1.16
	Tertiary education	1.01	0.94–1.08
**Labor status**			
	Employed or self-employed	1.07	0.99–1.15
	Unemployed	0.98	0.87–1.10
	Student	0.96	0.87–1.05
	Not in the labor force (retired, inactive)	0.94	0.86–1.03
**Net monthly income range, (€)**			
	1–1000	1.66	1.48–1.87
	1001–2000	0.78	0.69–0.98
	2001–3000	0.78	0.68–0.91
	3001–4000	0.80	0.64–0.99
	≥4001	0.85	0.66–1.12

^a^OR: odds ratio.

^b^EU: European Union.

From the viewpoint of residence and nationality, residence in other European Union countries (OR 1.28, 95% CI 1.09–1.50), and residence (OR 1.25, 95% CI 0.90–1.73) or birth (OR 1.31, 95% CI 1.06–1.61) outside the European Union determined higher probabilities of intensive eHealth usage. In contrast, European Internet users had a lower propensity toward more intensive eHealth usage if they had citizenship (OR 0.78, 95% CI 0.68–0.91) or were born in 1 of the 13 countries in the sample (OR 1.02, 95% CI 0.89–1.17). By municipality type, eHealth usage was more intensive among users residing in densely populated areas, such as cities and large towns (OR 1.17, 95% CI 1.09–1.25). Internet users residing in intermediate areas, such as towns (OR 0.92, 95% CI 0.86–0.99), or in less densely populated areas, such as village and rural areas (OR 0.90, 95% CI 0.83–0.97), had a lower propensity toward intensive eHealth usage.

Finally, European Internet users’ educational levels and occupational category presented an inverted U shape in relation to more intensive eHealth usage. Regarding levels of completed education, the propensity toward intensive eHealth usage was greater among those with a secondary education (OR 1.08, 95% CI 1.01–1.16). In contrast, users with primary (OR 0.87, 95% CI 0.80–0.95) and tertiary (OR 1.01, 95% CI 0.94–1.08) education had a lower propensity. In terms of occupational category, the propensity toward intensive eHealth usage was greater among the employed or self-employed (OR 1.07, 95% CI 0.99–1.15). Users who were unemployed (OR 0.98, 95% CI 0.87–1.10), students (OR 0.96, 95% CI 0.87–1.05), or not in the labor force (OR 0.94, 95% CI 0.86–1.03) had lower probabilities of more intensive eHealth usage. In explaining more intensive eHealth usage as a consequence of users’ net monthly income, the results suggest a higher propensity among the minimum wage stratum, earning ≤€1000 per month (OR 1.66, 95% CI 1.48–1.87).

## Discussion

The widespread use of ICTs in general and of the Internet in particular, together with the economic and social changes arising therefrom, are creating a fast-paced and significant change in relationships formed among the stakeholders of the health care system. One of the main manifestations of this disruptive process of change is the watering down of the traditional doctor-patient relationship model. Health Internet (eHealth) usage creates new dynamics that put the patient at the heart of the health care process. Doctor-patient interaction is no longer limited to time and place or to a few minutes in a doctor’s office; nowadays, digital flows of information, communication, and knowledge go beyond the scope of health care centers and pervade the daily lives of citizens.

In this new context, the importance of evaluating the extent to which eHealth usage empowers citizens and involves them in their health status has been noted in the literature [30,39]. While there is considerable evidence in the literature about the predictors of some particular uses of eHealth, generally for population samples [41], attention has recently been drawn to the need to use more advanced methods and models to evaluate the participation of patients and citizens in the shared health care model that eHealth proposes [36].

This is why the goal of our study was to design and evaluate a predictive multidimensional model of eHealth usage, comprising 9 dimensions and 88 indicators. To that end, we used a broad sample of 13,000 European Internet users. Although we did not use a population sample, the results obtained are very useful, for two reasons. First, obtaining new evidence centered solely on Internet users allowed us to focus the analysis better, particularly with regard to inequalities (health status, sex, age, nationality, territory, education, and occupational category) that determine intensive eHealth usage. Second, the predictors we obtained provided evidence that complements studies that have taken a population approach.

### eHealth Usage Composite Indicator

In recent years, eHealth usage has increased considerably [27,51]. More than half of the European population uses the Internet to look for health information [52], and more and more people are using it to access and manage their own personal health records [53], to buy health products and services, to communicate with their physicians [8,54], and to create digital content. In our study, we constructed a composite indicator using a 2-stage SEM methodology, and the results obtained are consistent with this evidence: they showed that, in 2011, 52.39% (6811/13,000) of European Internet users’ eHealth usage was intensive (higher than the mean). The dimensions with more explanatory power in the eHealth usage composite indicator were health Internet attitudes, information health Internet usage, empowerment of health Internet users, and usefulness of health Internet usage.

### Health Status-Related Predictors of eHealth

Regarding eHealth predictors, while differences between European Internet users’ perceived general health status and more intensive eHealth usage were not significant, long-term health problems or illnesses in the user or a family member did determine predictive power. European Internet users with long-term health problems or illnesses or receiving long-term treatment, or who had family members or cared for people with long-term health problems or illnesses had a greater propensity toward more intensive eHealth usage. Likewise, the study also highlighted that the existence of certain illnesses among the European Internet user population had high explanatory power with respect to intensive eHealth usage. These health problems or illnesses were diabetes, stroke or cerebral hemorrhage, cancer, and cataract. In contrast, users with health problems or illnesses related to chronic bronchitis and emphysema, and to osteoporosis had a lower propensity toward intensive eHealth usage.

These results, which are clearly consistent with other studies of social networking sites, virtual communities, and support group usage by patients with chronic illnesses [55], point to these patients’ need for information and communication flows via eHealth to manage their health problems. The link between eHealth and chronic health problems determines the choice to develop specific practices in this field, and especially to provide those in this segment of the population (the chronically ill and caregivers) who are still not Internet users with greater digital competencies.

### Sociodemographic-Related Predictors of eHealth

Our results suggest that women, those aged 25–54 years, and households with more members, more children <16 years of age, and more members >65 years of age were most likely to use eHealth intensively. In contrast, men, people in the age groups 16–24 years and 54–74 years, and households with fewer members or with fewer dependents were less likely to use eHealth intensively.

The decisive importance of women [44], the middle age segments, and care of dependents is explained by the nature of health care in households and by the progressive aging of the population. It is important to underscore that women’s role as health caregivers in the household clearly determines the usefulness of eHealth practices. In this respect, practices for fostering eHealth usage should consider the sex dimension more carefully. To a large extent, household eHealth usage arises through the health care role that families assign to women.

Aging of the population poses a broad set of challenges for health care systems, which a more widespread implementation of eHealth could help to meet. Without doubt, the main challenge for sustainable health that Europe faces over the coming years is the aging of the population. This is a complex mix of genetic, environmental, lifestyle, and socioeconomic factors, with the rates of associated chronic illnesses. Indeed, the European population is changing dramatically because of longer life expectancy and lower fertility rates. The number of European citizens over the age of 80 years is expected to double by 2025, which will give rise to increasingly complex needs in terms of clinical care, health care, and social care. In this context, eHealth practices could become one of the main tools for delivering health care to older citizens, especially through female caregivers. While the new patient-centered model has increasingly underscored the empowerment of patients and users in health care, the aging care model should be characterized by interaction between an active and informed patient or caregiver and a proactive and versatile medical team [56,57]. To that end, and given that the results obtained from this study show that middle-aged Internet users had a high propensity toward eHealth usage, it is essential to provide older caregivers who are still not Internet users with greater digital competencies.

From the perspective of nationality and territory, significant results were also obtained from the study. European Internet users had a greater propensity toward more intensive eHealth usage if they resided in other European Union countries or outside the European Union, and if they were born outside the European Union. Similarly, European Internet users’ residence in densely populated areas (cities or large towns) also better predicted eHealth usage. In this context, a fairer promotion of eHealth usage in Europe should also consider the territorial dimension, with special emphasis on connecting national health systems and a greater Internet presence and usage in less densely populated areas.

Finally, the results obtained also provide us with significant information about educational, occupational, and income categories, which are crucial for redressing some of the social inequalities in eHealth usage. Users’ educational levels explain more intensive eHealth usage, in an inverted U form. Thus, users with a secondary education had a greater propensity toward intensive eHealth usage. In this sense, the study provides new evidence (beyond population studies) in relation to middle-educated (secondary education) Internet users, who perceived the usefulness of eHealth usage. The education dimension also determines a new area of health inequality, and hence the need to promote Internet usage among the less educated population. The results related to occupational and income categories suggest a higher propensity among the employed or self-employed and among the minimum wage stratum earning ≤€1000 per month. Users who were integrated into the labor market, whether self-employed or employed, clearly had a greater propensity, whereas those who were not (students, unemployed, and not in the labor force) had a lower propensity to use eHealth. In this context, in order to achieve a more equitable eHealth usage, Internet usage among groups not actively integrated into the labor market should be promoted more vigorously. Regarding income, and in order to overcome inequalities, promoting eHealth usage skills (especially through education and learning) for workers with lower wages would also be very useful.

### Limitations

Our study has several limitations. First, there was a time lag between the year we obtained the data and the year we wrote the paper. However, we felt that the availability of a single database of 13,000 Internet users in Europe deserved an analysis despite the time lag. In future research, and as they become available, we will use newer data and introduce dynamic comparisons. Second, the study provides information only from the perspective of health users. In the future, we intend to address the issue of eHealth usage by health professionals. By doing so, we will be able to improve our multidimensional approach and obtain results and conclusions for all actors involved in eHealth usage. Third, the empirical methodology could also be improved by looking at the intensity of eHealth usage (not simply usage or mean usage) and at a higher number of predictors.

### Conclusions

The results obtained highlight the need for more in-depth research to be conducted into the link between eHealth usage and predictors, and the different health care systems in Europe. By doing so, it will be possible to increase the resolution of our results and to establish whether the intensity of eHealth usage varies depending on the health care systems, or the extent to which health care systems determine the prediction of eHealth usage. Similarly, strategic and public policy actions resulting from the research could be adapted more precisely to each health care system. Finally, the study results could be supplemented by the construction of a composite indicator of eHealth usage by health care professionals. The design, validation, and prediction of composite indicators of eHealth usage that take into consideration the perspectives of both users (ie, patients) and professionals in the different European health care systems would provide us with a very comprehensive view of the issue and would allow us to round off our multidimensional approach. We shall focus our efforts on all of these approaches in the near future.

## Appendix

Multimedia Appendix 1 Statistical information based on SIMPHS2 online survey.

Multimedia Appendix 2 Strategic Intelligence Mapping on Personal Health Systems Phase 2 (SIMPHS2) Questionnaire.

Multimedia Appendix 3 Health Internet uses descriptive statistics and frequency statistics. 2011.

Multimedia Appendix 4 Health care Internet uses descriptive statistics and frequency statistics. 2011.

Multimedia Appendix 5 Drivers of health care Internet uses and frequency statistics. 2011.

Multimedia Appendix 6 Barriers of health care Internet uses descriptive statistics and frequency statistics. 2011.

Multimedia Appendix 7 Usefulness of health Internet uses descriptive statistics and frequency statistics. 2011.

Multimedia Appendix 8 ICT uses descriptive statistics and frequency statistics. 2011.

Multimedia Appendix 9 Information health Internet uses descriptive statistics and frequency statistics. 2011.

Multimedia Appendix 10 Health Internet attitudes descriptive statistics and frequency statistics. 2011.

Multimedia Appendix 11 Empowerment of health Internet user’s descriptive statistics and frequency statistics. 2011.

## Abbreviations

CFI: comparative fit index
ICT: information and communication technology
IFI: incremental fit index
NFI: normed fit index
OR: odds ratio
RFI: relative fit index
RMSEA: root mean square error of approximation
SEM: structural equation modelling
SIMPHS2: Strategic Intelligence Monitor on Personal Health Systems Phase 2
TLI: Tucker-Lewis index
