# Supplementary material for: Modelling and Predicting eHealth Usage in Europe: A Multidimensional Approach From an Online Survey of 13,000 European Union Internet Users
Source: J Med Internet Res. 2016 Jul 22;18(7):e188. doi: 10.2196/jmir.5605 (PMC4975796; doi:10.2196/jmir.5605)
Supplement: Multimedia Appendix 1 [file jmir_v18i7e188_app1.pdf]

Appendix I. Statistical information based on SIMPHS2 online survey

Population	Citizens aged from 16 to 74 years old who have used the Internet in the last three months.
Scope of countries	Austria, Belgium, Germany, Denmark, Estonia, Finland, France, Italy, Netherlands, Sweden, Slovenia, Slovakia and Spain.
Type of survey	Online
Sample size	1,000 interviews per country.<br>13,000 interviews in total.
Quotas	Country<br>Gender (Female/Male)<br>Age Group (16-24/ 25-54/ 55-74)
Sampling error	±0.85% for overall data and ±3.16% for country-specific data. In all cases, a maximum indeterminate probability (p=q=50), for a confidence level of 95.5% is applicable for each one of the reference populations
Weighting	Proportional allocation for each country.<br>Weighting by country to be able to interpret the overall data.
Sampling	Individuals has been sampled in a completely random manner.

Source: SIMPHS2.
