# Supplementary material for: Modelling and Predicting eHealth Usage in Europe: A Multidimensional Approach From an Online Survey of 13,000 European Union Internet Users
Source: J Med Internet Res. 2016 Jul 22;18(7):e188. doi: 10.2196/jmir.5605 (PMC4975796; doi:10.2196/jmir.5605)
Supplement: Multimedia Appendix 2 [file jmir_v18i7e188_app2.pdf]

Appendix 2. Strategic Intelligence Mapping on Personal Health Systems Phase 2 (SIMPHS2) Questionnaire

We are currently conducting an International research study on behalf of the Institute for Prospective Technological Studies (IPTS), one of the seven scientific institutes of the European Commission's Joint Research Centre (JRC). The objective of the study is to analyze the use of Information and Communication Technologies (ICT), specially the Internet, for healthcare purposes. In this regard, we would like to ask for 20 minutes of your time to complete this survey. We would very much appreciate your opinion.

Please rest assured the survey is anonymous and the data gathered strictly confidential.

Block A: Health status and health care and social care services use

A1. How many times did you visit a doctor during the last 12 months? (include hospitalisation or visits to the outpatient department; do not include visits to the dentist)

***Number:*______________**	A1

A2. How many times have you received a doctor or a nurse at home during the last 12 months?

***Number:*______________**	A2

A3. How many times have did you visit or received a visit of a social care worker during the last 12 months?

***Number:*______________**	A3

A4. How is your health in general?

	A4
Very good	5
Good	4
Neither good or bad	3
Bad	2
Very bad	1

A5. Do you have any long-standing illness or health problem?

	A5
Yes	1
No	2
Don’t Know	99

A6. Are you undergoing a long-term medical treatment?

	A6
Yes	1
No	2
Don’t Know	99

A7. Over the past 6 months, to what extent, if at all, have you been limited in activities people normally do, because of a health problem. Would you say you have been…?

	A7
Severely limited	1
Somewhat limited	2
Not limited at all	3

A8. Do you have or have you ever had any of the following health problems?

	Yes	No	
Diabetes	1	2	A8_1
An allergy	1	2	A8_2
Asthma	1	2	A8_3
Hypertension (high blood pressure)	1	2	A8_4
Long-standing troubles with your muscles, bones and joints (rheumatism, arthritis)	1	2	A8_5
Cancer	1	2	A8_6
Cataract	1	2	A8_7
Migraine or frequent headaches	1	2	A8_8
Chronic bronchitis, emphysema	1	2	A8_9
Osteoporosis	1	2	A8_10
Stroke, cerebral haemorrhage	1	2	A8_11
Peptic ulcer (gastric or duodenal ulcer)	1	2	A8_12
Chronic anxiety or depression	1	2	A8_13

A9. Is someone close to you, currently experiencing long-term illness or disability?

	A9
Yes	1
No	2
Don’t know	99

If A9 = 1 -> A10
If A9 =2 or A9 =99 -> A11

A10. Are you taking care of such a person?

	A10
Yes	1
No	2

A11. In general, how often does your usual source of care (doctor or nurse)…

	Always	Often	Sometimes	Rarely	Never	
...explain to you the results of medical exams (laboratory, radiology, etc.)?	5	4	3	2	1	A11_1
...explain to you different treatment options?	5	4	3	2	1	A11_2
...listen to your opinion and take your preferences into account to choose treatments?	5	4	3	2	1	A11_3

A12. In general, how often do you ask your usual source of care (doctor or nurse)…

	Always	Often	Sometimes	Rarely	Never	
… to explain to you the results of the medical exams?	5	4	3	2	1	A12_1
… to explain to you the different treatment options?	5	4	3	2	1	A12_2
… to consider your opinion and your preferences when choosing treatments?	5	4	3	2	1	A12_3

Block B: Health attitude and Health information sources

B1. For each of the following statements regarding the use of Information and Communication Technologies, specially the Internet, could you please tell me whether you agree or disagree?

Information and Communication Technologies, specially the Internet, allow me to…

	Totally agree	Somewhat agree	Neither agree nor disagree	Somewhat disagree	Totally disagree	
be better informed about how to follow the advice of the physician or professionals I consult	5	4	3	2	1	B1_1
develop a better understanding of my personal health or that of a family member or friend by giving me access to recognized expert knowledge	5	4	3	2	1	B1_2
become better informed on what is available, such as the available solutions and treatments, so that I can make my own choices	5	4	3	2	1	B1_3
better understand my personal health or that of a family member or friend through my ability to determine what is relevant	5	4	3	2	1	B1_4
know more about the opinions of people who are in similar situations or who are active in support groups	5	4	3	2	1	B1_5
better understand my personal health or that of a family member or friend through online discussions or the opinions of people going through similar experiences	5	4	3	2	1	B1_6
play a more active role in my exchanges with my physician or the health professionals I consult	5	4	3	2	1	B1_7

B2. For each of the following statements regarding the use of Information and Communication Technologies, specially the Internet, could you please tell me whether you agree or disagree? ICT, specially the Internet, helps me feel …

	Totally agree	Somewhat agree	Neither agree nor disagree	Somewhat disagree	Totally disagree	
better equipped to implement the advice of the physician or health professionals I consult	5	4	3	2	1	B2_1
better equipped to make my own choices, without being limited to the advice of a physician or health professionals, which I believe is the best approach	5	4	3	2	1	B2_2
better equipped to make positive changes to my situation or that of a family member or friend through discussions and exchanges with others (in my family, at work, on the Internet, etc.)	5	4	3	2	1	B2_3
more confident in playing a more active role in my exchanges with my physician or the health professionals I consult	5	4	3	2	1	B2_4
more confident about the choices I plan on making, on my own, between the various possible treatments and solutions	5	4	3	2	1	B2_5
more confident in my discussions with the people in my life (my family, people at work or on the Internet, etc.)	5	4	3	2	1	B2_6

B3. For each of the following statements regarding the use of Information and Communication Technologies, specially the Internet, could you please tell me whether you agree or disagree? ICT, specially the Internet, facilitates…

	Totally agree	Somewhat agree	Neither agree nor disagree	Somewhat disagree	Totally disagree	
making decisions on my health albeit without going against the advice of the physician or the health professionals I have consulted	5	4	3	2	1	B3_1
a more active role in my health by deciding which solutions I prefer, whether from mainstream medicine or alternative approaches	5	4	3	2	1	B3_2
making decisions about my health on the basis of my preferences and means rather than only on the advice of my physician	5	4	3	2	1	B3_3
a more active role in my health by continuing to talk with the people in my life who could help me clarify my ideas	5	4	3	2	1	B3_4
making decisions about my health by relying on the experiences and points of view of the people with whom I talk (on the Internet, at work, in my family, etc.)	5	4	3	2	1	B3_5

B4. Below you can find a list of various sources of information about health, illness or wellness, and we would like to know how important these are to you.

	Very important	Somewhat important	Not so important	Not important at all	
Internet	4	3	2	1	B4_1
TV	4	3	2	1	B4_2
Radio	4	3	2	1	B4_3
Books, medical encyclopaedias and leaflets	4	3	2	1	B4_4
Courses and lectures	4	3	2	1	B4_5
Newspapers, magazines	4	3	2	1	B4_6
Family, friends and colleagues	4	3	2	1	B4_7
Pharmacies	4	3	2	1	B4_8
Direct face-to-face contact with doctors	4	3	2	1	B4_9
Direct face-to-face contact with nurses	4	3	2	1	B4_10

B5. Different authorities (government departments, local authorities, agencies) and private companies could offer health information and online services related with your health. To what extent do you trust the following institutions to protect your personal information?

	Trust fully	Trust somewha t	Trust little	Do not trust	
National public authorities (e.g. tax authorities, social security authorities)	4	3	2	1	B5_1
European institutions (European Commission, European Parliament, etc.)	4	3	2	1	B5_2
Banks and financial institutions	4	3	2	1	B5_3
Health and medical institutions	4	3	2	1	B5_4
Shops and department stores	4	3	2	1	B5_5
Internet companies (Search Engines, Social Networking Sites, E-mail Services)	4	3	2	1	B5_6
Phone companies, mobile phone companies and Internet Services Providers	4	3	2	1	B5_7
Pharmaceutical companies	4	3	2	1	B5_8

Block C: Internet and Information and Communication Technologies, uses

C1. Could you tell me if…?

	Every day or almost every day	At least once a week (but not every day)	At least once a month (but not every week)	Less than once a month	Never	
You use the Internet in your home	5	4	3	2	1	C1_1
You use the Internet at your place of work	5	4	3	2	1	C1_2
You use the Internet somewhere else (school, university, cyber-café, etc.)	5	4	3	2	1	C1_3

C2. Which of the following Internet related activities have you already carried out?

	Every day or almost every day	At least once a week (but not every day)	At least once a month (but not every week)	Less than once a month	Never	
Use a search engine to find information	5	4	3	2	1	C2_1
Send e-mails with attached files (documents, pictures, etc.)	5	4	3	2	1	C2_2
Post messages to chatrooms, newsgroups or an online discussion forum	5	4	3	2	1	C2_3
Use the Internet to make telephone calls	5	4	3	2	1	C2_4
Use peer-to-peer file sharing for exchanging movies, music, etc	5	4	3	2	1	C2_5
Create a web page	5	4	3	2	1	C2_6
Use websites to share pictures, videos, movies, etc.	5	4	3	2	1	C2_7
Use a social networking site	5	4	3	2	1	C2_8
Purchase goods or services online / online shopping (e.g. travel & holiday, clothes, books, tickets, films, music, software, food)	5	4	3	2	1	C2_9
Keep a blog (also known as web-log)	5	4	3	2	1	C2_10
Instant messaging, chat websites	5	4	3	2	1	C2_11
Do home banking	5	4	3	2	1	C2_12
Use online software	5	4	3	2	1	C2_13
Use the Internet through your mobile phone	5	4	3	2	1	C2_14
Online gaming and/or playing games console	5	4	3	2	1	C2_15

Block D: Health related use of Information and Communication Technologies, and the Internet

D1a. Regarding health, wellness and the Internet, how often have you….?

	Every day or almost every day	At least once a week (but not every day)	At least once a month (but not every week)	Less than once a month	Never	I was not aware of it	
looked for information about a physical illness or condition that you or someone you know has	5	4	3	2	1	9	D1a_1
looked for information about wellness or lifestyle	5	4	3	2	1	9	D1a_2
bought medicine or vitamins online	5	4	3	2	1	9	D1a_3
participated in an online support group for people who are concerned about the same health or medical issue	5	4	3	2	1	9	D1a_4
participated in Social Networking Sites talking about health and wellness	5	4	3	2	1	9	D1a_5
used email or gone to a web site to communicate with a doctor or a doctor's office	5	4	3	2	1	9	D1a_6
clicked on a health or medical web site's privacy policy to read about how the site uses personal information	5	4	3	2	1	9	D1a_7
described a medical condition or problem online in order to get advice from an online doctor	5	4	3	2	1	9	D1a_8
described a medical condition or problem online in order to get advice from other online users (peers)	5	4	3	2	1	9	D1a_9
kept a health web site "bookmarked", or saved as a "favourite place", so you can go back to it regularly	5	4	3	2	1	9	D1a_10
looked to see what company or organization is providing the advice or information that appears on a health web site	5	4	3	2	1	9	D1a_11
looked for information about a mental health issue like depression or anxiety	5	4	3	2	1	9	D1a_12
disclosed medical information on Social Networking Sites	5	4	3	2	1	9	D1a_13
disclosed medical information on websites to share pictures, videos, movies, etc.	5	4	3	2	1	9	D1a_14

For each reply where D1a_x=1 or 9 do the same for D1b_x

If D1a_1 = 1 and D1a_2= 1 -> D10

If D1a_1 = (2 to 5) or D1a_2 = (2 to 5) ->D2

D1b. Assuming that you were provided the possibility, state how likely it is that you would do the following during the next year?

	Very likely			Very unlikely	
look for information about a physical illness or condition that you or someone you know has	4	3	2	1	D1b_1
look for information about wellness or lifestyle	4	3	2	1	D1b_2
buy medicine or vitamins online	4	3	2	1	D1b_3
participate in an online support group for people who are concerned about the same health or medical issue	4	3	2	1	D1b_4
participate in Social Networking Sites talking about health and wellness	4	3	2	1	D1b_5
use email or gone to a web site to communicate with a doctor or a doctor's office	4	3	2	1	D1b_6
click on a health or medical web site's privacy policy to read about how the site uses personal information	4	3	2	1	D1b_7
describe a medical condition or problem online in order to get advice from an online doctor	4	3	2	1	D1b_8
describe a medical condition or problem online in order to get advice from other online users (peers)	4	3	2	1	D1b_9
keep a health web site "bookmarked", or saved as a "favourite place", so you can go back to it regularly	4	3	2	1	D1b_10
look to see what company or organization is providing the advice or information that appears on a health web site	4	3	2	1	D1b_11
look for information about a mental health issue like depression or anxiety	4	3	2	1	D1b_12
disclose medical information on Social Networking Sites	4	3	2	1	D1b_13
disclose medical information on websites to share pictures, videos, movies, etc.	4	3	2	1	D1b_14

D2. Were you looking for health and/or wellness information for yourself or for others? (multiple choice)

	Yes	No	
Yourself	1	2	D2_1
Child	1	2	D2_2
Parent	1	2	D2_3
Another relative	1	2	D2_4

Someone else	1	2	D2_5

If D2_1 = 1 -> D3

If D2_2 =1 or D2_3=1 or D2_4=1 or D2_5=1 ->D4

D3. Did you happen to go looking for this health information for yourself…?

	D3
Before visiting a doctor or clinic	1
After visiting a doctor or clinic	2
Instead of visiting a doctor or clinic	3
Unrelated to visiting a doctor or clinic	4

D4. Did you happen to go looking for this health information for another person…?

	D4
Before visiting a doctor or clinic	1
After visiting a doctor or clinic	2
Instead of visiting a doctor or clinic	3
Unrelated to visiting a doctor or clinic	4

D5. Overall, how USEFUL was the health information you got online

	D5
Very useful	4
Somewhat useful	3
Not too useful	2
Not at all useful	1

D6. Did you learn anything NEW from the information you got online, or not?

	D6
Yes	1
No	2
Don’t know	99

D7. Did you later talk to a doctor or nurse about the information you got online?

	D7
Yes	1
No	2
Don’t know	99

D8. Did the information you got online affect any of your decisions about health treatments or the way you take care of yourself?

	D8
Yes	1
No	2

D9. Did the information you got online affect the way you eat or exercise?

	D9
Yes	1
No	2
Don’t know	99

D10a. Regarding health and Information and Communication Technologies, specially the Internet, how often have you….?

	Every day or almost every day	At least once a week (but not every day)	At least once a month (but not every week)	Less than once a month	Never	I was not aware of it	
Made, cancelled or changed an appointment with your family doctor, specialist or other health professionals online	5	4	3	2	1	9	D10_1
Sent or received an email from your doctor, nurse or health care organization	5	4	3	2	1	9	D10_2
Made an online consultation through videoconference with your doctor or nurse	5	4	3	2	1	9	D10_3
Received online the results of your clinical or medical test.	5	4	3	2	1	9	D10_4
Accessed or uploaded your (or any other family member) medical information or health record through an Internet provider (ex. Google Health, Microsoft Vault…)	5	4	3	2	1	9	D10_5
Accessed or uploaded your (or any other family member) medical information or health record through an Internet application provided by your healthcare organization	5	4	3	2	1	9	D10_6
Used a game console to play games related with your health or your wellness	5	4	3	2	1	9	D10_7
Used a health/wellness application on your mobile phone	5	4	3	2	1	9	D10_8
Used devices (as pulse meter, glucose meter…) to transmit vital signs or other clinical information and/or received alarms or follow-up about your health anytime, anywhere	5	4	3	2	1	9	D10_9
Received any message about health promotion and/or health prevention	5	4	3	2	1	9	D10_10

For each reply where D10a_x=1 or 9 do the same for D10b_x

D10b. Assuming that you were provided the possibility, state how likely it is that you would do the following during the next year?

	Very likely			Very unlikely	
Make, cancel or change an appointment with your family doctor, specialist or other health professionals online	4	3	2	1	D10b_1
Send or receive an email from your doctor, nurse or health care organization	4	3	2	1	D10b_2
Make an online consultation through videoconference with your doctor or nurse	4	3	2	1	D10b_3
Receive online the results of your clinical or medical test.	4	3	2	1	D10b_4
Access or upload your medical information or health record through an Internet provider (ex. Google Health, Microsoft Vault…)	4	3	2	1	D10b_5
Access or upload your medical information or health record through Internet application provided by your healthcare organization	4	3	2	1	D10b_6
Use a game console to play games related with your health or your wellness	4	3	2	1	D10b_7
Use a health/wellness application on your mobile phone	4	3	2	1	D10b_8
Use devices (as pulse meter, glucose meter…) to transmit vital signs or other clinical information and/or received alarms or follow-up about your health anytime, anywhere	4	3	2	1	D10b_9
Receive any message about health promotion and/or health prevention	4	3	2	1	D10b_10

D11. Regardless of whether you have used Information and Communication Technologies for healthcare or wellness purposes, can you tell me how important you believe the following uses of Information and Communication Technologies and the Internet for heath or wellness purposes might be?

	Very important	Somewhat important	Not so importan t	Not important at all	
To prevent diseases by adopting a healthier lifestyle	4	3	2	1	D11_1
To obtain different points of view from those offered by mainstream medicine	4	3	2	1	D11_2
To better understand a health problem or disease	4	3	2	1	D11_3
To find a specific solution to or treatment for a health problem	4	3	2	1	D11_4
To find additional sources of information (addresses, references or links)	4	3	2	1	D11_5
To participate in online discussions	4	3	2	1	D11_6
To develop one's general knowledge or satisfy one's curiosity	4	3	2	1	D11_7
To help a family member or friend who is ill	4	3	2	1	D11_8
To access an online health service	4	3	2	1	D11_9

D12. Regardless of whether you have used Information and Communication Technologies or the Internet for healthcare or wellness purposes, would you tell us how important the following factors are when evaluating an internet health site?

	Very important	Somewha t important	Not so importan t	Not important at all	
Secure handling of personal information	4	3	2	1	D12_1
Information in my own language	4	3	2	1	D12_2
Updated information	4	3	2	1	D12_3
Interactivity, e.g. Question-and-answer service, discussion groups, chat	4	3	2	1	D12_4
Health professionals are involved	4	3	2	1	D12_5
Clearly stated who is responsible for sponsoring the site	4	3	2	1	D12_6
Health organizations are involved	4	3	2	1	D12_7
Governments are involved	4	3	2	1	D12_8

D13. Regardless of whether you have used Information and Communication Technologies for healthcare or wellness purposes, would you tell us how important the following barriers are in using these technologies for health or wellness purposes?

	Very important	Somewhat important	Not so importan t	Not important at all	
Lack of digital skills	4	3	2	1	D13_1
Lack of access to ICT for health applications	4	3	2	1	D13_2
Lack of motivation and interest	4	3	2	1	D13_3
Lack of awareness	4	3	2	1	D13_4
Lack of health literacy	4	3	2	1	D13_5
Lack of trust	4	3	2	1	D13_6
Lack of liability	4	3	2	1	D13_7
Lack of privacy	4	3	2	1	D13_8
Lack of security	4	3	2	1	D13_9
Lack of reliability	4	3	2	1	D13_10

D14. Assuming that you were provided the possibility of looking for health information on the Internet, would information on health or illness which you had obtained from the Internet lead to any of the following?

	Yes	No	Do not know	
Feelings of anxiety	1	2	99	D14_1
Feelings of reassurance or relief	1	2	99	D14_2
Willingness to change diet or other lifestyle habits	1	2	99	D14_3
Suggestions or queries on diagnosis or treatment to your family doctor, specialist or other health professional	1	2	99	D14_4
Changing of use of medicine without consulting your family doctor, specialist or other health professional	1	2	99	D14_5
Making, cancelling or changing an appointment with family doctor, specialist or other health professional	1	2	99	D14_6

D15. To what extent do you agree with the following statements?

	Totally agree	Somewhat agree	Neither agree nor disagree	Somewhat disagree	Totally disagree	
ICT for health could increase my use of the ICT in other fields of my daily life	5	4	3	2	1	D15_1
ICT for health could lead to greater patients satisfaction	5	4	3	2	1	D15_2
ICT for health could improve my health status	5	4	3	2	1	D15_3
ICT for health could improve the ability to take care and monitor my own health	5	4	3	2	1	D15_4
ICT for health could change my behaviours towards a healthy lifestyle	5	4	3	2	1	D15_5
ICT for health could avoid travelling expenses and time	5	4	3	2	1	D15_6
ICT for health could improve the quality of health care services received	5	4	3	2	1	D15_7
Internet health services substitute some of my face-to-face consultations with the physicians	5	4	3	2	1	D15_8
Internet health services complement some of my face-to-face consultations with the physicians	5	4	3	2	1	D15_9
The quality of Internet health services is aligned with the quality of face-to-face services	5	4	3	2	1	D15_10
I have concerns about the kind of personal information shared with physicians or health organizations through the Internet due to privacy and confidentiality issues	5	4	3	2	1	D15_11
In case of need, I would feel more comfortable and safe at home with a remote monitoring system to track my health	5	4	3	2	1	D15_12
I would be willing to pay to access Internet health services for myself or my relatives	5	4	3	2	1	D15_13

Block E: Socio demographic profile of participants

E1. Gender

	E1
Male	1
Female	2

E2. How old are you?

Age:_____________	E2

E3. Which is your country of citizenship?

	E3
National to UK	1
National of other EU member state	2
National of non-EU country	3

E4. Which is your country of birth?

	E4
UK Native	1
Born in another EU member state	2
Born in non-EU country	3

E5. What is your highest level of education completed?

	E5
Primary or lower secondary education [ISCED 0,1 or 2]	1
Upper secondary education [ISCED 3 or 4]	2
Tertiary education [ISCED 5 or 6]	3

E6. Which of these descriptions best describes your situation or applies to what you have been doing for the last month?

	E6
Employed or self-employed (incl. family workers)	1
Unemployed	2
Student (not in the labour force)	3
Other not in the labour force<br>(retired, inactive, in compulsory military service, etc.)	4

If E6= 1 -> E7

If E6 = (2 to 4) -> E8

E7.- What is your occupation?

____E7

(Recoded into at least 2-digit ISCO-88 categories)

E8. Region of residence:

Description:________(Recoded)	E8

E9. Type of locality:

	E9
Densely-populated area (Cities and Large towns)	1
Intermediate area (Towns)	2
Thinly-populated area (Villages and Rural)	3

E10. Number of members in the household?

Number:___________	E10

E11. Of which, number of children under 16 years?

Number:___________	E11

E12. Of which, number of members over 65 years?

Number:___________	E12

E13. Which is your average net monthly income?

	E13
GBP: _________________	1
Do not want to answer	99
