# Supplementary material for: Modelling and Predicting eHealth Usage in Europe: A Multidimensional Approach From an Online Survey of 13,000 European Union Internet Users
Source: J Med Internet Res. 2016 Jul 22;18(7):e188. doi: 10.2196/jmir.5605 (PMC4975796; doi:10.2196/jmir.5605)
Supplement: Multimedia Appendix 3 [file jmir_v18i7e188_app3.pdf]

Appendix 3a. Health Internet uses descriptive statistics. 2011

	N	Mean	Std. Dev.	Minimum	Maximum	Skewness	Kurtosis
1. Look for information about a physical illness (LINFPHILL)	12,555	2.46	1.025	1	5	0.537	-0.219
2. Look for information about wellness or lifestyle (LINFWLFS)	12,488	2.48	1,105	1	5	0.402	-0.592
3. Buy medicine or vitamins online (BMEDVIT)	11,498	1.52	0.939	1	5	1.939	3.170
4. Participate in an online support group with people (POLSUPGR)	11,390	1.55	0.989	1	5	1.866	2.647
5. Participate in Social Networking Sites (PSOCNET)	11,608	1.64	1.031	1	5	1.630	1.815
6. Use email or web to communicate with a doctor's office (UMWCODR)	11,410	1.62	0.989	1	5	1.717	2.338
7. Click on a health or medical web's privacy policy (CLWPRIPO)	11,308	1.68	1.043	1	5	1.527	1.496
8. Describe a medical condition to get advice from an online doctor (DMDCOLDR)	11,401	1.52	0.952	1	5	1.988	3.331
9. Describe a medical condition to get advice from other online users (DMDCOLUS)	11,681	1.59	0.977	1	5	1.754	2.406
10. Keep a health web site bookmarked or saved as a favourite place (BKMHWS)	11,902	1.96	1.181	1	5	1.043	0.020
11. Look to see what company is providing the information on a health website (LCOPHIN)	11,890	1.91	1.064	1	5	1.086	0.441
12. Look for information about a mental health issue (LINFMHI)	11,897	1.83	1.067	1	5	1.240	0.753
13. Disclose medical information on Social Networking Sites (DSNETMDINF	11,335	1.43	0.930	1	5	2.264	4.382
14. Disclose medical information on websites to share files (DWMEDINF)	11,254	1.40	0.920	1	5	2.414	4.989

Source: Own elaboration.

Appendix 3b. Health Internet uses frequency statistics. 2011

		Valid percentage*				
	N	1	2	3	4	5
1. Look for information about a physical illness (LINFPHILL)**	12,555	16.3	41.7	25.8	12.4	3.8
2. Look for information about wellness or lifestyle (LINFWLFS)**	12,488	20.9	33.7	26.5	14.5	4.4
3. Buy medicine or vitamins online (BMEDVIT)**	11,498	69.8	16.3	8.0	4.0	1.9
4. Participate in an online support group with people (POLSUPGR)**	11,390	70.3	14.4	8.1	5.1	2.2
5. Participate in Social Networking Sites (PSOCNET)**	11,608	64.3	18.1	9.4	5.6	2.6
6. Use email or web to communicate with a doctor's office (UMWCODR)**	11,410	63.5	20.8	8.7	4.5	2.5
7. Click on a health or medical web's privacy policy (CLWPRIPO)**	11,308	61.5	19.8	10.3	5.8	2.6
8. Describe a medical condition to get advice from an online doctor (DMDCOLDR)**	11,401	70.5	15.8	7.4	4.1	2.2
9. Describe a medical condition to get advice from other online users (DMDCOLUS)**	11,681	65.6	19.0	8.4	4.8	2.1
10. Keep a health web site bookmarked or saved as a favourite place (BKMHWS)**	11,902	49.9	22.2	14.5	9.1	4.3
11. Look to see what company is providing the information on a health website (LCOPHIN)**	11,890	46.1	29.5	14.7	7.0	2.8
12. Look for information about a mental health issue (LINFMHI)**	11,897	51.5	26.4	12.5	6.7	2.9
13. Disclose medical information on Social Networking Sites (DSNETMDINF)**	11,335	77.4	10.0	6.7	3.9	2.1
14. Disclose medical information on websites to share files (DWMEDINF)**	11,254	80.2	8.0	5.7	4.2	2.0

* 1=Never; 2=Less than once a month; 3=At least once a month, but not every week; 4=At least once a week, but not every day; 5=Every day or almost every day.

** Missing= I was not aware of it (1=445; 2=512; 3=1,502; 4=1,610; 5=1,392; 6=1,590; 7=1,692; 8=1,599; 9=1,319; 10=1,098; 11=1,110; 12=1,103; 13=1,665; 14=1,746).

Source: Own elaboration.
