# Supplementary material for: Modelling and Predicting eHealth Usage in Europe: A Multidimensional Approach From an Online Survey of 13,000 European Union Internet Users
Source: J Med Internet Res. 2016 Jul 22;18(7):e188. doi: 10.2196/jmir.5605 (PMC4975796; doi:10.2196/jmir.5605)
Supplement: Multimedia Appendix 4 [file jmir_v18i7e188_app4.pdf]

Appendix 4a. Health care Internet uses descriptive statistics. 2011

	N	Mean	Std. Dev.	Minimum	Maximum	Skewness	Kurtosis
15. Internet appointment with health care professionals (INAPPHP)	12,305	1.50	0.886	1	5	2.054	4.020
16. Emailing from doctor, nurse or health care organization (EMAILHP)	12,336	1.46	0.854	1	5	2.192	4.735
17. Online consultation through videoconference with health care professionals (OLVIDHP)	11,962	1.23	0.738	1	5	3.509	11.949
18. Receive online the results of clinical or medical test (ROLMEDR)	12,164	1.34	0.808	1	5	2.716	7.156
19. Use of Medical information through an Internet provider (MEDINFINPRO)	11,877	1.28	0.787	1	5	3.076	8.980
20. Use of Medical information through an Internet health care organization (MEDINFINHO)	11,983	1.28	0.782	1	5	3.023	8.724
21. Game console to play games related with health or wellness (PGAMEH)	12,083	1.37	0.874	1	5	2.526	5.761
22. Use of Health/wellness application on mobile phone (MAPPH)	12,117	1.31	0.826	1	5	2.862	7.605
23. Use electronic devices to transmit clinical or medical information (EDEMEDINF)	12,168	1.42	0.934	1	5	2.356	4.770
24. Emailing about health promotion and/or health prevention (EMAILHPR)	12,366	1.63	0.993	1	5	1.640	2.010

Source: Own elaboration.

Appendix 4b. Health care Internet uses frequency statistics. 2011

		Valid percentage*				
	N	1	2	3	4	5
15. Internet appointment with health care professionals (INAPPHP)**	12,305	68.3	20.4	6.3	3.3	1.8
16. Emailing from doctor, nurse or health care organization (EMAILHP)**	12,336	70.4	19.6	5.3	3.1	1.6
17. Online consultation through videoconference with health care professionals (OLVIDHP)**	11,962	89.4	3.4	3.5	2.3	1.3
18. Receive online the results of clinical or medical test (ROLMEDR)**	12,164	80.5	10.7	4.5	2.9	1.4
19. Use of Medical information through an Internet provider (MEDINFINPRO)**	11,877	86.3	5.4	4.1	2.9	1.4
20. Use of Medical information through an Internet health care organization (MEDINFINHO)**	11,983	85.2	6.5	4.3	2.7	1.3
21. Game console to play games related with health or wellness (PGAMEH)**	12,083	80.5	8.6	5.7	3.4	1.8
22. Use of Health/wellness application on mobile phone (MAPPH)**	12,117	84.3	6.5	4.4	3.2	1.6
23. Use electronic devices to transmit clinical or medical information (EDEMEDINF)**	12,168	78.4	9.7	5.6	4.1	2.3
24. Emailing about health promotion and/or health prevention (EMAILHPR)**	12,366	62.9	20.6	9.3	5.0	2.2

* 1=Never; 2=Less than once a month; 3=At least once a month, but not every week; 4=At least once a week, but not every day; 5=Every day or almost every day.

** Missing= I was not aware of it (15=695; 16=664; 17=1,038; 18=836; 19=1,123; 20=1,017; 21=917; 22=883; 23=832; 24=634).

Source: Own elaboration.
