# Supplementary material for: Modelling and Predicting eHealth Usage in Europe: A Multidimensional Approach From an Online Survey of 13,000 European Union Internet Users
Source: J Med Internet Res. 2016 Jul 22;18(7):e188. doi: 10.2196/jmir.5605 (PMC4975796; doi:10.2196/jmir.5605)
Supplement: Multimedia Appendix 5 [file jmir_v18i7e188_app5.pdf]

Appendix 5a. Drivers of health care Internet uses descriptive statistics. 2011

	N	Mean	Std. Dev.	Minimum	Maximum	Skewness	Kurtosis
25. Secure handling of personal information (SECHANPINF)	13,000	3.60	0.724	1	4	-1.898	3.092
26. Information in own language (INFOLAN)	13,000	3.46	0.783	1	4	-1.401	1.281
27. Updated Information (UPDINF)	13,000	3.52	0.733	1	4	-1.584	2.155
28. Interactivity (INTERACT)	13,000	2.78	0.891	1	4	-0.281	-0.681
29. Health professionals are involved (HPINV)	13,000	3.40	0.778	1	4	-1.257	1.090
30. Clearly stated who is responsible for sponsoring the site (CLEARWS)	13,000	2.98	0.967	1	4	-0.536	-0.779
31. Health organizations are involved (HORGINV)	13,000	3.12	0.828	1	4	-0.699	-0.095
32. Governments are involved (GOVINV)	13,000	2.54	0.964	1	4	-0.025	-0.964

Source: Own elaboration.

Appendix 5b. Drivers of health care Internet uses frequency statistics. 2011

		Valid percentage*			
	N	1	2	3	4
25. Secure handling of personal information (SECHANPINF)	13,000	2.7	6.2	19.7	71.5
26. Information in own language (INFOLAN)	13,000	3.1	9.0	26.5	61.5
27. Updated Information (UPDINF)	13,000	2.7	6.2	27.3	63.7
28. Interactivity (INTERACT)	13,000	8.5	27.4	41.4	22.6
29. Health professionals are involved (HPINV)	13,000	3.3	8.4	33.0	55.4
30. Clearly stated who is responsible for sponsoring the site (CLEARWS)	13,000	8.8	21.6	32.8	36.8
31. Health organizations are involved (HORGINV)	13,000	4.5	15.6	43.3	36.6
32. Governments are involved (GOVINV)	13,000	15.7	32.9	33.0	18.5

*1=Not important at all; 2=Not so important; 3=Somewhat important; 4=Very important.

Source: Own elaboration.
