# Supplementary material for: Modelling and Predicting eHealth Usage in Europe: A Multidimensional Approach From an Online Survey of 13,000 European Union Internet Users
Source: J Med Internet Res. 2016 Jul 22;18(7):e188. doi: 10.2196/jmir.5605 (PMC4975796; doi:10.2196/jmir.5605)
Supplement: Multimedia Appendix 6 [file jmir_v18i7e188_app6.pdf]

Appendix 6a. Barriers of health care Internet uses descriptive statistics. 2011

	N	Mean	Std. Dev.	Minimum	Maximum	Skewness	Kurtosis
33. Lack of digital skills (LKESKIL)	13,000	2.83	0.941	1	4	-0.421	-0.705
34. Lack of access to ICT for health applications (LKICTHAPP)	13,000	2.96	0.867	1	4	-0.542	-0.359
35. Lack of motivation and interest (LKMOTINT)	13,000	3.01	0.830	1	4	-0.557	-0.231
36. Lack of awareness (LKAWRNSS)	13,000	3.09	0.840	1	4	-0.691	-0.113
37. Lack of health literacy (LKHLTRCT)	13,000	3.12	0.848	1	4	-0.718	-0.138
38. Lack of trust (LKTRUST)	13,000	3.29	0.805	1	4	-0.999	0.452
39. Lack of liability (LKLIABLT)	13,000	3.19	0.839	1	4	-0.815	-0.007
40. Lack of privacy (LKPRIVCT)	13,000	3.32	0.817	1	4	-1.059	0.428
41. Lack of security (LKSECURT)	13,000	3.34	0.803	1	4	-1.081	0.544
42. Lack of reliability (LKREABLT)	13,000	3.32	0.788	1	4	-1.056	0.628

Source: Own elaboration.

Appendix 6b. Barriers of health care Internet uses frequency statistics. 2011

		Valid percentage*			
	N	1	2	3	4
33. Lack of digital skills (LKESKIL)	13,000	10.7	22.3	40.7	26.3
34. Lack of access to ICT for health applications (LKICTHAPP)	13,000	6.7	19.5	44.6	29.2
35. Lack of motivation and interest (LKMOTINT)	13,000	5.1	18.6	46.3	30.0
36. Lack of awareness (LKAWRNSS)	13,000	5.1	15.8	43.7	35.4
37. Lack of health literacy (LKHLTRCT)	13,000	5.0	15.8	41.8	37.4
38. Lack of trust (LKTRUST)	13,000	3.7	10.9	38.0	47.3
39. Lack of liability (LKLIABLT)	13,000	4.4	14.3	39.4	41.9
40. Lack of privacy (LKPRIVCT)	13,000	3.6	11.5	33.7	51.2
41. Lack of security (LKSECURT)	13,000	3.4	10.8	34.4	51.4
42. Lack of reliability (LKREABLT)	13,000	3.4	10.0	37.5	49.1

*1=Not important at all; 2=Not so important; 3=Somewhat important; 4=Very important.

Source: Own elaboration.
