# Supplementary material for: Modelling and Predicting eHealth Usage in Europe: A Multidimensional Approach From an Online Survey of 13,000 European Union Internet Users
Source: J Med Internet Res. 2016 Jul 22;18(7):e188. doi: 10.2196/jmir.5605 (PMC4975796; doi:10.2196/jmir.5605)
Supplement: Multimedia Appendix 7 [file jmir_v18i7e188_app7.pdf]

Appendix 7a. Usefulness of health Internet uses descriptive statistics. 2011

	N	Mean	Std. Dev.	Minimum	Maximum	Skewness	Kurtosis
43. ICT for health could increase other ICT uses (EHICTL)	13,000	3.23	1.129	1	5	-0.369	-0.430
44. ICT for health could lead to greater patient's satisfaction (EHPATSAT)	13,000	3.51	1.062	1	5	-0.567	-0.032
45. ICT for health could improve health status (EHHSAT)	13,000	3.24	1.102	1	5	-0.389	-0.345
46. ICT for health could improve the ability to take care own health (EHMONH)	13,000	3.50	1.073	1	5	-0.583	-0.077
47. ICT for health could change behaviours towards a healthy lifestyle (EHBEHS)	13,000	3.41	1.072	1	5	-0.503	-0.154
48. ICT for health could avoid travelling expenses and time (EHAVOEXT)	13,000	3.61	1.119	1	5	-0.634	-0.162
49. ICT for health could improve the quality of health care services (EHIMQHC)	13,000	3.50	1.071	1	5	-0.548	-0.106
50. Internet health substitute of-line consultations with the physicians (EHSUBOLHC)	13,000	2.79	1.271	1	5	0.013	-1.066
51. Internet health complements of-line consultations with the physicians (EHCOMOLHC)	13,000	3.42	1.166	1	5	-0.561	-0.404
52. Quality of Internet health is aligned with the quality of of-line services (EHQAOLHCQ)	13,000	2.94	1.144	1	5	-0.105	-0.671
53. Personal information shared with physicians though Internet due to privacy (EHINFPPRCON)	13,000	3.58	1.140	1	5	-0.522	-0.421
54. More comfortable with a remote monitoring system to track health (EHCOMONH)	13,000	3.15	1.214	1	5	-0.302	-0.757
55. Willing to pay to access Internet health services (EHPAY)	13,000	2.53	1.246	1	5	0.241	-1.001

Source: Own elaboration.

Appendix 7b. Usefulness of health care Internet uses frequency statistics. 2011

		Valid percentage*				
	N	1	2	3	4	5
43. ICT for health could increase other ICT uses (EHICTL)	13,000	10.5	10.9	36.5	29.6	12.6
44. ICT for health could lead to greater patient's satisfaction (EHPATSAT)	13,000	6.2	7.9	31.9	36.6	17.5
45. ICT for health could improve health status (EHHSAT)	13,000	9.6	11.0	36.9	30.8	11.8
46. ICT for health could improve the ability to take care own health (EHMONH)	13,000	6.5	8.6	30.4	37.5	17.1
47. ICT for health could change behaviours towards a healthy lifestyle (EHBEHS)	13,000	7.1	9.4	33.5	35.3	14.7
48. ICT for health could avoid travelling expenses and time (EHAVOEXT)	13,000	6.4	7.8	27.4	34.6	23.7
49. ICT for health could improve the quality of health care services (EHIMQHC)	13,000	6.2	8.6	31.4	36.1	17.6
50. Internet health substitute of-line consultations with the physicians (EHSUBOLHC)	13,000	22.2	17.4	28.6	22.5	9.3
51. Internet health complements of-line consultations with the physicians (EHCOMOLHC)	13,000	9.6	9.7	27.5	35.8	17.4
52. Quality of Internet health is aligned with the quality of of-line services (EHQAOLHCQ)	13,000	14.2	17.3	37.3	22.4	8.7
53. Personal information shared with physicians though Internet due to privacy (EHINFPPRCON)	13,000	6.1	10.2	27.9	31.4	24.4
54. More comfortable with a remote monitoring system to track health (EHCOMONH)	13,000	13.6	12.9	31.5	28.6	13.4
55. Willing to pay to access Internet health services (EHPAY)	13,000	29.0	18.5	29.3	16.6	6.5

* 1=Totally disagree; 2=Somewhat disagree; 3=Neither agree nor disagree; 4=Somewhat agree; 5=Totally agree.

Source: Own elaboration.
