# Supplementary material for: Modelling and Predicting eHealth Usage in Europe: A Multidimensional Approach From an Online Survey of 13,000 European Union Internet Users
Source: J Med Internet Res. 2016 Jul 22;18(7):e188. doi: 10.2196/jmir.5605 (PMC4975796; doi:10.2196/jmir.5605)
Supplement: Multimedia Appendix 8 [file jmir_v18i7e188_app8.pdf]

Appendix 8a. ICT uses descriptive statistics. 2011

	N	Mean	Std. Dev.	Minimum	Maximum	Skewness	Kurtosis
56. Use a search engine to find information (FINDINF)	13,000	4.54	0.798	1	5	-2.123	5.009
57. Send e-mails with attached files (EMAILATTF)	13,000	4.02	1.063	1	5	-0.975	0.237
58. Post messages to chatrooms, newsgroups or an online discussion forum (POSTMESS)	13,000	2.61	1.457	1	5	0.362	-1.271
59. Use the Internet to make telephone calls (TELPCALL)	13,000	2.16	1.374	1	5	0.832	-0.694
60. Use peer-to-peer file sharing for exchanging pictures, videos or movies (FILESHARE)	13,000	1.99	1.325	1	5	1.057	-0.242
61. Create a web page (CREATEWP)	13,000	1.66	1.110	1	5	1.718	1.973
62. Use websites to share pictures, videos or movies (USWSHARE)	13,000	2.64	1.434	1	5	0.306	-1.275
63. Use a social networking site (USESNET)	13,000	3.40	1.569	1	5	-0.422	-1.386
64. Purchase goods or services online shopping (PURB2C)	13,000	2.75	1.016	1	5	0.355	-0.306
65. Keep a blog, web-log (KEEPBLOG)	13,000	1.68	1.174	1	5	1.628	1.422
66. Instant messaging, chat websites (CHATMESS)	13,000	2.87	1.550	1	5	0.109	-1.510
67. Home banking (HBANKING)	13,000	3.43	1.283	1	5	-0.714	-0.531
68. Use online software (USEESOFT)	13,000	2.75	1.402	1	5	0.229	-1.239
69. Use the Internet through mobile phone (USEINMOBP)	13,000	2.41	1.598	1	5	0.579	-1.301
70. Online gaming and/or playing games console (EGAME)	13,000	2.62	1.514	1	5	0.333	-1.390

Source: Own elaboration.

Appendix 8b. ICT uses frequency statistics. 2011

		Valid percentage*				
	N	1	2	3	4	5
56. Use a search engine to find information (FINDINF)	13,000	1.4	1.8	5.9	23.6	67.4
57. Send e-mails with attached files (EMAILATTF)	13,000	2.8	7.7	15.9	32.1	41.5
58. Post messages to chatrooms, newsgroups or an online discussion forum (POSTMESS)	13,000	32.2	20.9	15.5	16.0	15.4
59. Use the Internet to make telephone calls (TELPCALL)	13,000	48.1	18.3	12.1	12.5	9.0
60. Use peer-to-peer file sharing for exchanging pictures, videos or movies (FILESHARE)	13,000	55.0	16.0	11.2	10.3	7.6
61. Create a web page (CREATEWP)	13,000	65.0	17.9	7.1	5.5	4.5
62. Use websites to share pictures, videos or movies (USWSHARE)	13,000	31.2	19.8	17.2	17.6	14.1
63. Use a social networking site (USESNET)	13,000	20.7	11.8	11.5	18.8	37.2
64. Purchase goods or services online shopping (PURB2C)	13,000	8.8	34.5	35.5	15.2	6.1
65. Keep a blog, web-log (KEEPBLOG)	13,000	68.2	12.0	8.1	6.6	5.0
66. Instant messaging, chat websites (CHATMESS)	13,000	29.4	17.0	13.4	17.8	22.3
67. Home banking (HBANKING)	13,000	14.9	5.8	20.0	39.9	19.5
68. Use online software (USEESOFT)	13,000	25.4	22.3	19.4	17.5	15.3
69. Use the Internet through mobile phone (USEINMOBP)	13,000	47.5	12.7	9.4	12.4	18.1
70. Online gaming and/or playing games console (EGAME)	13,000	35.4	17.6	13.1	17.1	16.7

* 1=Never; 2=Less than once a month; 3=At least once a month, but not every week; 4=At least once a week, but not every day; 5=Every day or almost every day.

Source: Own elaboration.
