# Supplementary material for: Modelling and Predicting eHealth Usage in Europe: A Multidimensional Approach From an Online Survey of 13,000 European Union Internet Users
Source: J Med Internet Res. 2016 Jul 22;18(7):e188. doi: 10.2196/jmir.5605 (PMC4975796; doi:10.2196/jmir.5605)
Supplement: Multimedia Appendix 9 [file jmir_v18i7e188_app9.pdf]

Appendix 9a. Information health Internet uses descriptive statistics. 2011

	N	Mean	Std. Dev.	Minimum	Maximum	Skewness	Kurtosis
71. Better informed about the advice of the health care professionals (ADVHCP)	13,000	3.79	1.022	1	5	-0.787	0.349
72. Better understanding of personal health (UNDPH)	13,000	3.93	0.983	1	5	-0.933	0.741
73. Better informed on what is available, so that can make own choices (AVOWNHCH)	13,000	3.98	0.979	1	5	-0.976	0.796
74. Better understand personal health, through to determine their relevance (UNDREPH)	13,000	3.85	0.985	1	5	-0.815	0.507
75. Know more about the opinions of people who are in similar situations (KNOWPPH)	13,000	3.95	1.006	1	5	-0.934	0.628
76. Better understand personal health through online discussions or experiences (UNDPHTP)	13,000	3.74	1.064	1	5	-0.740	0.120
77. Play a more active role in exchanges with health care professionals (PLAYACTRO)	13,000	3.63	1.079	1	5	-0.623	-0.058

Source: Own elaboration.

Appendix 9b. Information health Internet uses frequency statistics. 2011

		Valid percentage*				
	N	1	2	3	4	5
71. Better informed about the advice of the health care professionals (ADVHCP)	13,000	4.0	5.6	24.0	39.8	26.6
72. Better understanding of personal health (UNDPH)	13,000	3.2	4.5	19.9	41.6	30.9
73. Better informed on what is available, so that can make own choices (AVOWNHCH)	13,000	2.9	4.4	18.6	40.4	33.8
74. Better understand personal health, through to determine their relevance (UNDREPH)	13,000	3.2	5.1	22.9	41.5	27.3
75. Know more about the opinions of people who are in similar situations (KNOWPPH)	13,000	3.4	4.2	20.6	38.0	33.8
76. Better understand personal health through online discussions or experiences (UNDPHTP)	13,000	4.7	6.8	24.5	37.9	26.1
77. Play a more active role in exchanges with health care professionals (PLAYACTRO)	13,000	5.4	7.4	28.5	35.7	23.0

* 1=Totally disagree; 2=Somewhat disagree; 3=Neither agree nor disagree; 4=Somewhat agree; 5=Totally agree.

Source: Own elaboration.
