# Supplementary material for: Modelling and Predicting eHealth Usage in Europe: A Multidimensional Approach From an Online Survey of 13,000 European Union Internet Users
Source: J Med Internet Res. 2016 Jul 22;18(7):e188. doi: 10.2196/jmir.5605 (PMC4975796; doi:10.2196/jmir.5605)
Supplement: Multimedia Appendix 10 [file jmir_v18i7e188_app10.pdf]

Appendix 10a. Health Internet attitudes descriptive statistics. 2011

	N	Mean	Std. Dev.	Minimum	Maximum	Skewness	Kurtosis
78. Better equipped to implement the advice of the health care professionals (BEQADHP)	13,000	3.74	1.003	1	5	-0.768	0.426
79. Better equipped to make own choices without to the advice of a physician (BEQOWNCH)	13,000	3.62	1.075	1	5	-0.623	-0.082
80. Better equipped to make positive changes through other people (BEQCHTDISP)	13,000	3.73	1.002	1	5	-0.708	0.305
81. More confident in playing a more active role in physician's relationship (MCONPLACTRL)	13,000	3.71	1.023	1	5	-0.696	0.205
82. More confident about the choices on possible treatments and solutions (MCONPTREAT)	13,000	3.74	1.018	1	5	-0.734	0.281
83. More confident in discussions with the people in my life (MCONDISPE)	13,000	3.73	1.027	1	5	-0.708	0.218

Source: Own elaboration.

Appendix 10b. Health Internet attitudes frequency statistics. 2011

		Valid percentage*				
	N	1	2	3	4	5
78. Better equipped to implement the advice of the health care professionals (BEQADHP)	13,000	4.1	5.7	25.2	42.3	22.8
79. Better equipped to make own choices without to the advice of a physician (BEQOWNCH)	13,000	5.2	8.4	27.2	37.2	21.9
80. Better equipped to make positive changes through other people (BEQCHTDISP)	13,000	3.8	6.0	26.5	40.8	22.9
81. More confident in playing a more active role in physician's relationship (MCONPLACTRL)	13,000	4.2	6.5	26.5	39.6	23.2
82. More confident about the choices on possible treatments and solutions (MCONPTREAT)	13,000	4.0	6.1	25.5	40.3	24.0
83. More confident in discussions with the people in my life (MCONDISPE)	13,000	4.2	6.1	26.4	39.1	24.1

* 1=Totally disagree; 2=Somewhat disagree; 3=Neither agree nor disagree; 4=Somewhat agree; 5=Totally agree.

Source: Own elaboration.
