# Supplementary material for: Modelling and Predicting eHealth Usage in Europe: A Multidimensional Approach From an Online Survey of 13,000 European Union Internet Users
Source: J Med Internet Res. 2016 Jul 22;18(7):e188. doi: 10.2196/jmir.5605 (PMC4975796; doi:10.2196/jmir.5605)
Supplement: Multimedia Appendix 11 [file jmir_v18i7e188_app11.pdf]

Appendix 11a. Empowerment of health Internet user's descriptive statistics. 2011

	N	Mean	Std. Dev.	Minimum	Maximum	Skewness	Kurtosis
84. Making decisions on health albeit without going against the physicians (HDECWTAHP)	13,000	3.62	1.073	1	5	-0.661	-0.006
85. More active role in health by deciding solutions or alternative approaches (ACTHROLE)	13,000	3.67	1.038	1	5	-0.678	0.124
86. Making decisions about health on the basis of own preferences (HDECWPREF)	13,000	3.54	1.096	1	5	-0.586	-0.205
87. More active role in health by continuing to talk with the people (ACTHROLETPE)	13,000	3.67	1.020	1	5	-0.657	0.178
88. Making decisions about health by relying on the experiences with the people (HDECWPE)	13,000	3.51	1.081	1	5	-0.563	-0.177

Source: Own elaboration.

Appendix 11b. Empowerment of health Internet user's frequency statistics. 2011

		Valid percentage*				
	N	1	2	3	4	5
84. Making decisions on health albeit without going against the physicians (HDECWTAHP)	13,000	5.5	8.0	26.7	38.6	21.2
85. More active role in health by deciding solutions or alternative approaches (ACTHROLE)	13,000	4.5	7.2	26.7	39.5	22.1
86. Making decisions about health on the basis of own preferences (HDECWPREF)	13,000	6.2	9.9	27.2	37.6	19.2
87. More active role in health by continuing to talk with the people (ACTHROLETPE)	13,000	4.3	6.7	28.1	39.7	21.2
88. Making decisions about health by relying on the experiences with the people (HDECWPE)	13,000	6.1	9.8	28.7	37.5	17.9

* 1=Totally disagree; 2=Somewhat disagree; 3=Neither agree nor disagree; 4=Somewhat agree; 5=Totally agree.

Source: Own elaboration.
